# Features of High-Precision Photothermal Analysis of Liquid Systems by Dual-Beam Thermal Lens Spectrometry

**DOI:** 10.3390/nano14191586

**Published:** 2024-10-01

**Authors:** Vladislav R. Khabibullin, Ivan V. Mikheev, Mikhail A. Proskurnin

**Affiliations:** 1 Analytical Chemistry Division, Chemistry Department, M.V. Lomonosov Moscow State University, d. 1, Str. 3, Lenin Hills, GSP-1 V-234, Moscow 119991, Russia; vladhab1995@gmail.com (V.R.K.); mikheev.ivan@gmail.com (I.V.M.); 2 Federal State Budgetary Institution of Science Institute of African Studies, Russian Academy of Sciences, St. Spiridonovka, 30/1, Moscow 123001, Russia

**Keywords:** thermal lens spectrometry, accuracy, precision, trueness, thermal diffusivity, absorbance-based photothermal signal, finely dispersed systems

## Abstract

Thermal lens spectrometry is a high-sensitivity method for measuring the optical and thermal parameters of samples of different nature. To obtain both thermal diffusivity and absorbance-based signal measurements with high accuracy and precision, it is necessary to pay attention to the factors that influence the trueness of photothermal measurements. In this study, the features of liquid objects are studied, and the influence of optical and thermal effects accompanying photothermal phenomena are investigated. Thermal lens analysis of dispersed solutions and systems with photoinduced activity is associated with a large number of side effects, the impact of which on trueness is not always possible to determine. It is necessary to take into account the physicochemical properties and optical and morphological features of the nanophase and components exhibiting photoinduced activity. The results obtained make it possible to reduce systematic and random errors in determining the thermal-diffusivity-based and absorbance-based photothermal signals for liquid objects, and also contribute to a deeper understanding of the physicochemical processes in the sample.

## 1. Introduction

Photothermal spectroscopy (PTS) is a group of methods based on recording the nonradiative relaxation of excited molecules. A special place among PTS is occupied by thermal lens spectrometry (TLS), which is based on recording changes in the refractive index of the medium and provides simultaneous analysis of the optical (absorbance and temperature coefficient of the refractive index) and thermal parameters (thermal diffusivity) of samples rapidly and without complex sample preparation. This provides great opportunities in the tests and analysis of a wide range of solid and liquid samples of various nature (aqueous and organo-aqueous solutions, finely dispersed and photochemical systems, biological samples, etc.) [1].

The dual nature of the recorded data in TLS requires increased attention to the issues of measurement accuracy. Identification and systematization of factors affecting the accuracy and precision of measurements of both thermal diffusivity and absorbance-based signals for such a multifactor method is challenging. In the practice of photothermal spectroscopy, there are studies on identifying and analyzing the quantitative influence of various factors (detector pinhole size [2], the noises caused by the radiation source and background absorption [3,4], influence of beam sizes [5], and other factors [6]) on the accuracy and precision of the results of photothermal measurements of simple homogeneous systems (aqueous and organic solutions of inert molecular colorants). Analysis and consideration of factors affecting the accuracy and precision for simple and single-component systems has provided the results of photothermal measurements for more or less chemically trivial qualitative and quantitative problems being in good agreement with the results of other methods, and in some cases surpass them in metrological parameters. The use of TLS in colorimetric analysis [7,8,9,10], chromatography and flow techniques [11,12,13,14], studies of chemical kinetics and thermodynamics [10,15,16,17], surfaces and thin films [18,19,20], and thermophysical analysis [21,22] has shown low systematic and random errors. However, for more complex systems, TLS demonstrates unacceptably low or at least questionable accuracy and/or precision of results [23,24,25].

First of all, a significant amount of photothermal data has accumulated, indicating the limitations of TLS in the studies of multicomponent and/or multiphase samples. Difficulties are observed in the analysis of finely dispersed systems and samples with photoinduced properties [24,26,27]. To solve current problems in materials science, sustainable energy sources, and environmental management, it is necessary to measure thermal diffusivity with high accuracy and record absorption in such systems [28,29,30,31].

At the same time, most conventional methods of optical absorption spectroscopy and thermophysical testing have a number of shortcomings in the assessment of thermal diffusivity; e.g., spectrophotometry has a high systematic error from light scattering and lower sensitivity (by 2–3 orders of magnitude) compared to TLS [10,32,33,34]. A spectrophotometer, however, is simpler in terms of instrumentation and does not require increased attention to adjustment (position of sample cells with the sample compartment, beam sizes in the sample, centering of the probe beam on the detector, etc.). Fluorescence spectroscopy is a more sensitive method, even more sensitive compared with TLS. However, it is applicable to a much smaller number of fluorescent samples, and fluorescence represents the radiation energy yield that competes with heat transfer [1,25], so this method is not applicable for thermal diffusivity. Instead, fluorescence spectroscopy and TLS are both used and complement each other in assessing radiative and non radiative quantum yields, showing similar accuracy [35].

Thermal diffusivity is assessed using a large number of optical and thermophysical methods. The most common thermophysical methods include pulsed electrothermal method [36], transition hot wire [37,38], 3ω method [39], and heat flow (or heat flux) method [40]. In thermophysical methods of analysis, thermal diffusivity is usually measured indirectly, through the ratio of thermal conductivity to heat capacity, density, viscosity, etc., which significantly complicates the analysis [41,42]; e.g., the 3ω method, which is based on electrothermal heating and is well suited for thin film analysis [43], has many deviations from the 2D model. Close attention must be paid to the purity, shape, and thickness of the heating wire [39]. In addition, the duration of the analysis complicates the registration of physicochemical processes occurring in colloidal and photodynamic systems. In addition, most thermophysical methods require complex sample preparation and a large amount of analyte, which limits the use of the method in microanalysis (e.g., in biological fluids). The heat flow method, which is successfully used in production and research tasks of analyzing large volumes of liquid and solid objects (glass, rubber, etc.) [40], has a number of disadvantages associated with heat loss, long signal recording time, and low sensitivity to the microcomponent composition of samples [44].

Optical methods for measuring thermal diffusivity include the laser flash method [45], stimulated Rayleigh scattering [46], thermal-wave interferometry [47], the laser intensity modulation method [48], and photothermal methods (photoacoustic spectroscopy, photothermal radiometry, mirage spectroscopy, and thermal lens spectrometry) [1,42]. Optical methods for measuring thermal diffusivity are more sensitive than thermophysical methods, but they have limitations associated with complex instrumentation; e.g., laser flash and thermal wave interferometry methods are designed to analyze opaque solid films and coatings and are sensitive to the geometric parameters of the sample and holder, heat loss (vacuuming is often used), and the power and time of the pulse [49,50,51]. In comparison with thermophysical methods, PTS has the necessary sensitivity in studying fine processes in systems, and simple instrumentation provides advantages in comparison with other optical methods of measuring thermal diffusivity. However, the lack of systematic studies of factors affecting the accuracy of photothermal measurements, as well as general approaches to result processing, do not allow photothermal spectroscopy methods to occupy a more significant place in the assessment of thermal and optical properties of complex systems.

The problem of accuracy of photothermal measurements of complex systems has previously been raised in a number of studies, in which solutions to specific or general issues of photothermal analysis were proposed. It has been shown [52] that setups with a broad beam waist (150–300 μm) provide a longer time to reach thermal equilibrium and, thus, better accuracy of thermal diffusivity measurements. In [44,53], the features of photothermal analysis of dispersed systems with different optical absorption were considered, as a result of which recommendations were proposed for processing and presenting the results of time-resolved measurements, making it possible to identify side thermal effects and reduce the systematic error in determining the thermal diffusivity. A systematization and analysis of the additive influence of instrumental factors (changes in the transverse mode of the excitation beam, sample-detector distance, positioning of the probe beam on the detector, etc.) on the trueness of photothermal measurements is presented [54].

However, a number of factors associated with the influence of thermal and optical effects, as well as with the peculiarities of the analysis of complex samples, have not yet been systematically considered. An approach to considering the sources of systematic and random errors for samples of complex composition should be based on comparison and generalization of the results of photothermal studies of a large number of different systems. Finding the error sources of photothermal analysis of complex samples will make it possible to establish conditions for measuring thermal diffusivity and thermal lens signals with low systematic and random errors, identify the limits of applicability of the method and develop methodological recommendations for the analysis of complex samples. Solving this problem may resolve general issues of the validity of photothermal analysis and allow moving on to a broader problem, the uncertainty of photothermal measurements.

This paper discusses factors affecting the accuracy and precision of thermal diffusivity and thermal lens signal measurements that are associated with the test sample. Special attention is paid to finely dispersed systems and the analysis of samples with photoinduced activity. In such systems, it is necessary to take into account the physicochemical nature of the test sample and the morphological and optical parameters of the analyte.

## 2. Materials and Methods

### 2.1. Reagents and Test Samples

The following solvents were used in the study: ethanol purchased from Merck (Darmstadt, Germany), and chloroform, acetonitrile, and toluene in chemical purity purchased from Ecos-1 (Moscow, Russia). Lithium chloride, potassium, and sodium, as well as sodium bromide and sodium iodide used in the work, were chemically pure and purchased from Reakhim (Moscow, Russia). Bovine serum albumin (BSA) was purchased from Dia-M (Moscow, Russia) with a purity of 99% (ca. 66 kDa, CAS 9048-46-8).

SiO_2_ nanoparticle dispersions of Ludox series (SM-30, HS-40, TM-50) purchased from Merck (Germany); the main parameters are presented in Table S1. The procedure for preparing working solutions is described in detail in [44].

Aqueous dispersions of graphene oxide (GO, Hummer’s type) were purchased from Rusgrafen (Moscow, Russia) and fractionated using a standard protocol published in [55]. The procedure for preparing working GO dispersions of various fractions is presented in detail in [53].

Aqueous dispersions of uncolored and colored polystyrene particles were used in this work with the operating parameters presented in Table S2. The conditions for the synthesis of latex particles are described in detail in [56,57].

Aqueous dispersions of Fe_3_O_4_ (magnetite) nanoparticles with an average size of 10 ± 3 nm were prepared by co-precipitation from a mixture of FeCl_2_ and FeCl_3_ solutions in a molar ratio of 1:2, described in detail in [58,59]. Working solutions were prepared by diluting the resulting dispersion with deionized water using a Sapphire ultrasonic bath (Moscow, Russia) for 1 h.

Aqueous solutions of BSA were prepared by dissolving a sample of the protein in deionized water using ultrasound for 1 h. Samples were stored for no more than 7 days at a temperature of +(4.0 ± 0.5) °C.

A solution of *p*-chlorophenoxy-substituted lutetium phthalocyanine in chloroform with a concentration of 10 nmol/L was prepared in accordance with the protocol proposed in [60]. Physicochemical properties are presented in detail in [61].

Mixing was carried out using a laboratory shaker model PE-6410, purchased from EKROSHIM (St. Petersburg, Russia).

### 2.2. Thermal Lens Measurements

**Figure 1:**
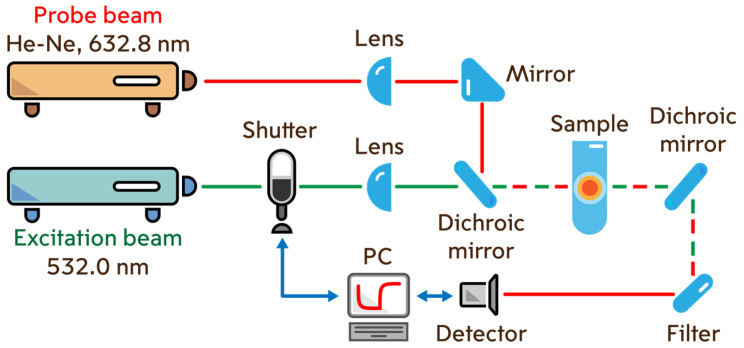
Dual-beam thermal lens spectrometer.

Photothermal measurements were carried out on a dual-beam thermal lens spectrometer, the scheme of which is shown in Figure 1. All spectrometer optical and mechanical components, unless otherwise noted, were purchased from ThorLabs (Newton, NJ, USA). The excitation beam was a solid-state laser (model MGL-FN-532, λ_e_ = 532 nm, TEM_00_ mode) purchased from Changchun New Industries Optoelectronics Tech. Co., Ltd. (Changchun, China). Radiation from a helium-neon laser (model HNL050L, λ_p_ = 632.8 nm, TEM_00_ mode) was used as a probe beam. A photodiode was used as a detector. A chopper (model SH05) was used to modulate the heating and cooling cycle. The heating and cooling cycles were controlled using an original program written in the C++ programming language (Borland Corp., Austin, TX, USA) on a personal computer through an analog-to-digital and digital-to-analog converter (model c8051Fx-DK) purchased from Silicon Labs (Boston, MA, USA). Laser power was measured using a power meter (Model Optronics Nova II) purchased from Ophir Optronics Solutions (Jerusalem, Israel).

**Figure 2:**
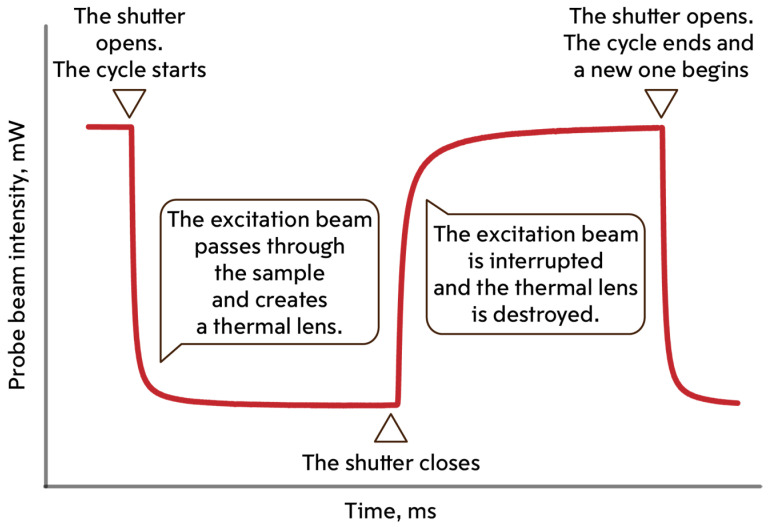
Signal of one cycle of time-resolved measurements.

The signal from the detector is recorded every cycle, continuously. A cycle begins when the modulator shutter opens, continues when it closes, and ends only when the shutter opens again (Figure 2). The PC records data in the form of intensity versus time from the detector.

When the modulator shutter opens, the excitation beam passes through a quartz cell (*l* = 10.00 mm) with a sample, in which it induces a temperature gradient acting as a thermal lens. The probe beam, passing through the heated area, expands. A drop in the intensity of the probe beam is recorded at the detector. When the shutter is lowered, the excitation beam is closed and heating of the sample stops. Cooling leads to relaxation of the thermal lens. The probe beam intensity increases. Measurement parameters are given in Table S3. A detailed description of the spectrometer operation is given elsewhere [44,54].

### 2.3. Other Measurements

Absorbance measurements and UV/vis spectra (*l* = 10.00 mm, cell volume 0.3 cm^3^) were measured using an Agilent Cary 4000 spectrophotometer purchased from Agilent (Mulgrave, Australia).

### 2.4. Finding the Thermal Lens Signal and Thermal Diffusivity

The behavior of a thermal lens can be described by parabolic or aberrant models using diffraction theory. Each of these approaches has its own advantages and disadvantages [62]. The parabolic model proposed by Gordon in 1965 [63] and further refined [64] describes the generic behavior of a thermal lens and is convenient to use. However, the temperature rise and change in refractive index for the excitation beam are not parabolic, and the thermal lens cannot be considered as an ideal thin optical lens [62,65]. In Sheldon’s aberrant model, the temperature increase has an integral form, and a change in the refractive index causes only a phase shift of laser radiation [66]. This model is more accurate compared to the parabolic. Moreover, both models are applicable only for a single-beam spectrometer or a dual-beam spectrometer with matched beam modes [62,64]. In 1992, based on the aberrant model of Sheldon, Shen and Snook developed a model to describe the thermal lens in the case of a dual-beam spectrometer with unmatched beam modes [65] (the model is also applicable for single-beam and double-beam mode-matched spectrometers). This model is suitable for describing the results of both stationary and time-resolved measurements [65]. At the same time, the model proposed by Shen and Snook (in one form or another) is currently used in solving most problems of photothermal spectroscopy (fluorescence [67], quantitative determination, thermal diffusivity analysis, etc.). For this reason, in this work, Shen–Snook’s model is used to describe the transient curves and find the thermal and optical properties.

According to the model [65], the intensity of the probe laser on the detector at the time of development of the thermo-optical element *I*(*t*) is presented as:

(1) $$I\left(t\right)=I\left(0\right){\left[1-\frac{\theta }{2}{tan}^{-1}\left(\frac{2mV}{\left[{\left(1+2m\right)}^{2}+V^{2}\right]\left(t_{c}/2t\right)+1+2m+V^{2}}\right)\right]}^{2},$$

which depends on the initial direction of the probe beam *I*(0) at time t=0, geometric parameters *m* and *V*, thermo-optical signal *θ* and characteristic time $t_{c}$. The geometric parameters *V* and *m* (mode-matching factor) are as follows:

(2) $$V=z_{1}/z_{c}+z_{c}/z_{2}\left[1+{\left(z_{1}/z_{c}\right)}^{2}\right],$$

and

(3) $$m=({\omega_{p1}/\omega_{e0})}^{2}$$

where $\omega_{p1}$ is the probe beam radius in the sample; $\omega_{e0}$ is the excitation-beam waist radius, in the sample; $z_{c}\;$ is the confocal distance for the probe laser; and $z_{1}\;$ and $z_{2}$ are the distances from the probe beam waist to the center of the cell with the sample and from the cell to the detector, respectively.

The thermo-optical signal *θ* depends on the excitation laser power, *P*; the linear absorption factor, α; the optical path length (cell size), *l*; thermal diffusivity, *k*; the excitation laser wavelength, *λ_e_*; and the temperature coefficient of the refractive index, d*n*/d*T*:

(4) $$\theta =P\alpha l/(k\lambda_{e})\cdot (-dn/dT),$$

The geometric parameters of the spectrometer and the thermo-optical signal, as can be seen, depend on previously known quantities (reference data or additional measurements). The characteristic time $t_{c}$, on the contrary, is an experimental parameter, which is individual for each “sample/spectrometer” system. Characteristic time $t_{c}$ is related to thermal diffusivity, *D*, according to:

(5) $$t_{c}=\omega_{e0}^{2}/4D,$$

which provides the assessment of thermal diffusivity and, thus, with external data, thermal conductivity and/or heat capacity.

The recorded thermal lens signal is a relative change in intensity:

(6) $$\vartheta =\frac{I\left(0\right)-I\left(\infty \right)}{I\left(\infty \right)}$$

Here, *I*(0) is the intensity of the probe beam at time *t* = 0; the averaging of the last 300 ms of the transient curve was used as the stationary state $I\left(\infty \right)$.

Shen–Snook’s model was developed for homogeneous true solutions where convection currents and thermal diffusion are absent [65]. Model limitations are [65]:

- The sample thickness is low compared to the confocal distances of the beams $z_{c}$ to ensure that the beam sizes are constant throughout the sample.
- To prevent edge effects, the surface of the sample is large compared to the area of the excitation beam in the sample, $\omega_{e0}$.
- The laser power absorbed by the sample is small and there is no convection flow.
- The change in refractive index with temperature d*n*/d*T* is constant with increasing sample temperature.

As well, in dispersed systems and solutions with photochemical transformations, there are side thermal and optical effects, and concentration and thermal diffusion. To measure the thermal diffusivity and thermal lens signal in such systems, an adaptation of the model was carried out, described in detail in [44,53]. According to the proposed approaches, Equation (1) can be represented as:

(7) $$I\left(t\right)=I\left(0\right){\left[1-0.5\theta {tan}^{-1}\left(a/\left(b\left(t_{c}/2t\right)+c\right)\right)\right]}^{2}\;,$$

where *a*, *b*, and *c* are the geometric constants of the spectrometer: *a* = 2*mV*, *b* = (1 + 2*m*)^2^ + *V*^2^, *c* = 1 + 2*m* + *V*^2^.

As shown by thermal lens studies of the dispersion of nanoparticles, solutions of proteins and macromolecules, quantum dots, etc., the characteristic time of the thermal lens depends on the parameters of the macro- and microcomponents of each system [68,69]. In this case, from Equation (7), the nanophase-dependent thermal lens characteristic time can be represented as a function of time:

(8) $$\tilde{t_{c}}\left(t\right)=[(a/tan[2\cdot (1-\sqrt{I\left(t\right)/I\left(\infty \right)})/\theta^{\prime }])-c]\cdot 2t/b,$$

where $\tilde{t_{c}}\left(t\right)$ is the effective characteristic time at each moment of the transition curve development. In the case of a true solution or in the absence of thermophoresis, the steady state $I\left(\infty \right)$ is understood as the averaged value of the last points of the transition curve [1]. In the case of thermophoresis and for heterogeneous systems, the corrected intensity of the probe beam $I^{\prime }\left(\infty \right)$, was used, which is obtained by fitting the first 100–150 ms of the transition curve in such a way that the last points of the experimental curve fully correspond to the theoretical one.

The transition from the effective $\tilde{t_{c}}$ to the true characteristic time $t_{c}$ occurs by averaging the values of the first 100–150 ms $\tilde{t_{c}}\left(t\right)$. Then, using Equation (5), the thermal diffusivity of the object is found. The proposed approach provides the systematic error in finding thermal parameters at a level below 5%.

## 3. Results and Discussion

**Figure 3:**
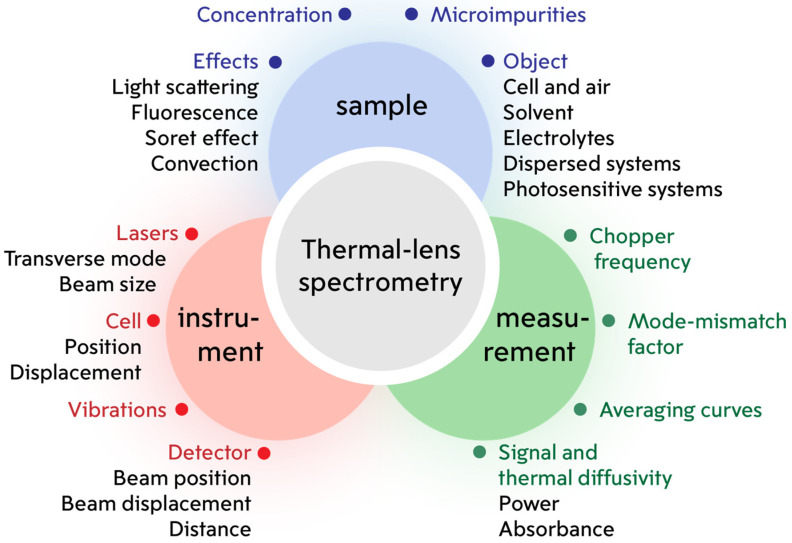
Scheme of factors influencing the accuracy and precision of measurements of thermal diffusivity and absorbance-based photothermal signals in thermal lens spectrometry.

This study of the influence of accuracy and precision factors concerns all thermal lens spectrometry without restrictions to a specific type of the setup. In the practice of thermal lens measurements, both single-beam and dual-beam instruments are used, operating in continuous or pulsed modes of generating an excitation beam. There is a sufficient number of studies related to the consideration of the advantages and disadvantages of each type of TLS setups [1,70,71]. Despite the configuration differences, the approaches used in recording and interpreting data, and analytical capabilities, different types of thermal lens spectrometers share similar factors that influence the measurement trueness. Figure 3 is a summary of major factors that may affect the accuracy and precision of TLS.

The main instrumental factors and factors associated with measurement parameters have been partially discussed previously [2,52,54,72,73]. These are well-studied sources of systematic and random error, the magnitude of which is not affected by the nature of the test sample. In this study, factors associated with the test sample and effects arising during measurements (Soret effect, light scattering, and convection) are studied. At the same time, the thermal and optical effects under consideration are well known and described [74,75,76], but their influence on the accuracy and precision of photothermal measurements has not been studied. The influence of the presence of electrolytes, the environment, and the nature of the solvent on photothermal measurements is also considered here.

Particular attention is paid to the accuracy of measurements of finely dispersed systems, solutions of proteins and some macromolecules with photoinduced activity. The experiments made in this study are discussed along with the data of previous publications that can be treated from the viewpoint of accuracy and precision of photothermal measurements.

### 3.1. Photothermal Effect of Quartz Cell and Air

Atmospheric air and cell material influence the results of photothermal measurements [77]. The walls of the cell and air, when a laser beam passes through them, are able to generate a thermal lens and contribute to the resulting signal [1,18,77]. As a rule, the contribution of the introduced signal is insignificant and is ignored in most models [1,4,62,65,66,78]. However, this factor also introduces an error, especially when assessing samples with low optical absorption.

The photothermal signal from a quartz cell with an optical path length of 10.00 mm is ca. 5 times higher than a signal from air (Table 1). The positive temperature coefficient of the refractive index for quartz accounts for the negative sign of the thermal lens signal [79]. For pulsed thermal lens spectrometry, the difference between glass and air was approximately four orders of magnitude [80]. This is due to the fact that the temperature change during pulsed laser excitation occurs much faster than the diffusion of heat into the environment. It takes longer for air to reach the maximum signal value than for glass. The heat diffusion from glass into air is a relatively slow process compared to the excitation.

For a thermal lens signal of 0.02 (deionized water at 300 mW), the introduced measurement error (under the same conditions) caused by the cell walls can be more than 5% [77]. As the signal increases (due to absorption or power), the error decreases and at *ϑ* > 0.15 the influence of the cell wall is <1%.

In addition to the photothermal effect in the cell walls, there is a problem of wall-to-sample heat transfer [77]. Photothermal measurements of the refractive index gradient, using a photothermal-deflection method, confirmed the thermal transfer of heat between the glass surface and the coupling fluid, which shows another shortcoming of the boundary conditions of the models usually assumed in TLS experiments [79]. This has a significant impact on the correctness of determination of the temperature change occurring inside the sample. The results showed that the glass exhibits large deviations due to the direct contact of the sample with air [79].

An important factor when working with quartz (or glass) cell is the degree of cleanliness of the windows. It is obvious that over time, nano- and microparticles, macromolecules, dyes, etc. are adsorbed on the windows, which contribute to the thermal lens signal and the dynamics of the development of transient curves [18]. In [72], using the example of TLS analysis of permanganate in tap water, it was shown that the signal introduced by MnO_2_ precipitated from tap water is significant and can be fivefold higher than the true value. This leads to decreased accuracy and an increased systematic error in quantitative analysis. In this regard, periodic physical and chemical cleaning of the cell is mandatory. Exposure to dilute acids (phosphoric, sulfuric, and hydrochloric acids) for 4–5 min removes 90% of adsorbed particles [72].

### 3.2. Light Scattering

Light scattering in turbid samples does not affect the accuracy and precision of thermal diffusivity measurements. A comparison of the absorbance measurement results of thermal lens spectrometry and optical spectroscopy (spectrophotometry) for the same turbid sample showed that in the first case the determination error did not exceed 5–10%, while for spectrophotometry the error exceeded 300% [33]. Attenuation of transmitted radiation due to light scattering by 15% gives an apparent absorbance of 0.07 units, which leads to poor accuracy for spectrophotometry, but not for TLS [81]. TLS gives fairly accurate absorbance values almost independent of the scattering coefficient.

In thermal lens spectrometry, light scattering can affect the signal value not only by attenuating the probe beam, similar to what is observed in the transmission measurements, but also by attenuating the excitation beam, which leads to a loss of power absorbed by the analyte [32,33,82]. It has been established that scattering does not affect the measured signal, provided that the dissipated power is small compared to the incident power and the spatial profile of the probe beam does not change [32,83]. Therefore, scattering losses <20% are acceptable in TLS [81].

The photothermal signal is well described by the predictions of the conventional thin- lens approximation for turbidity values below 6 cm^–1^ in a 1 cm-thick sample [84]. In this approximation, a Gaussian excitation beam profile and hence the induced thermal lens is assumed to be unaffected by scattering losses. However, at turbidity values greater than 7 cm^–1^, the photothermal signal starts to decrease sharply. Sufficiently high turbidities distort the field wavefront within the sample, decreasing temperature gradients and, as a consequence, reducing the strength of the thermal lens [84]. A turbidity of 8.6 cm^–1^ results in a 78-fold reduction in transmittance, resulting in only a 2.5-fold reduction in the photothermal signal. A turbidity of 15 cm^–1^ leads to a decrease in transmittance by more than 2000 times. In this case, the TLS signal decreases by only 20 times. The signal-to-noise ratio is 12 for a sample with a turbidity of 15 cm^–1^. High turbidity affects the thermal refractive gradient and distorts the profiles of both the exciting and probe beams. Up to turbidity values of the order of 6–7 cm^–1^, the thin lens approximation works quite well. No significant changes were observed at turbidity below 1 cm^–1^ [84].

### 3.3. Fluorescence

Fluorescence has a similar effect on the accuracy and precision of photothermal measurements as light scattering and the presence of fluorescence reduces the thermal lens signal [1,25]. This is mainly caused by a decrease in the thermo-optical effect due to the transfer of part of the absorbed energy in the form of radiation. The addition of a quencher (e.g., KI) improves the conversion efficiency of absorbed energy into thermal energy and thereby increases the thermal lens signal [85]. On the other hand, concentration and/or oxygen quenching do not increase the thermal lens signal [85]. Photothermal spectroscopy is actively used in fluorescence analysis [86]. The higher sensitivity of TLS in detecting dimerization processes and finding the quantum yield (*Φ*_f_) than classical fluorescence spectroscopy is emphasized [27,87,88,89]. Despite this, PTS has its limitations. TLS do not cope well with assessing low values of *Φ*_f_ [90].

For pulsed TLS, it has been shown previously that assessing the quantum yield with high accuracy is possible only in the range of 0.93 to 0.95 [90]. It has also been found that high powers of the excitation laser lead to optical saturation, which reduces fluorescence and non-radiative relaxation processes [90]. If optical saturation is present only at the fluorophore, the thermal lens signal of the fluorophore will be underestimated compared to the signal of the reference absorber, and the fluorescence quantum yield will be overestimated. Photothermal assessment of quenching also requires taking into account the salt effect, which affects the thermo-optical properties of the medium; e.g., in water, the thermal lens signal is enhanced due to the salt effect, and the quantum yield of fluorescence will be overestimated [90]. The study [91] revealed an error of 5% in finding the distance between Ag and Rhodamine 6G nanoparticles using TLS. For single-beam TLS using Sheldon’s model, there is a 5% uncertainty in finding the fluorescence quantum yield, which the authors associate with the selection of coefficients for the main equation of the transient curve [92]. At the same time, the authors point out the poor capabilities of the method in analyzing fluorescence in inhomogeneous samples [92].

For TLS with continuous laser radiation, studies on the analysis of fluorescence quenching [93] found that photothermal results are consistent with the results obtained by fluorescence spectroscopy only in a limited range of quencher concentrations. The amount of thermal energy released by the quenching reaction exceeded the associated radiative energy loss. The heat release for a non-fluorescent compound should be unity, but experimental fluorescence quenching values are higher than expected and can be greater than unity even if fluorescence quenching is not complete. These deviations are not only detrimental when using photothermal methods to study fluorescence quenching reactions, but are also an important drawback when determining absolute fluorescence quantum yields [93].

### 3.4. Convection and Mass Transfer

Excessive heating of the sample can cause convection to occur in the thermal lens region, leading to local changes in temperature and refractive index gradient. As a result, the probe beam undergoes deformation of the intensity profile and changes in direction, which deteriorates the results of photothermal measurements [32,94].

Convection and fluid flow (laminar or turbulent) influence the relative magnitude of the photothermal signal and generate noise [95]. At high excitation powers or background absorption, periodic wave-like changes in the signal are observed in the sample [96]. This can be mistakenly taken for changes in the laser intensity. However, with rigid localization of the rays in the sample (the position of the thermal lens), if periodic changes in the signal are observed, then these are convection effects in the sample. Much of this effect occurs in chromatographic analysis, where there is flow and there is a need for precise positioning of the beams in a thin capillary (or flow tube). Flow increases the effective thermal conductivity of the sample. As the flow rate increases, a decrease in the characteristic time is observed (which is consistent with an increase in the effective thermal conductivity) [97].

It is shown [98] that an ideal thermal lens spectrometer suitable for solving trivial problems in liquid chromatography should be based on a single laser with sufficient output power to cause a certain increase in the signal (a second laser only complicates the alignment and does not lead to a real reduction in noise). It is noted that a flow of several tens of microliters per minute does not affect the signal-to-noise ratio [98]. The influence of flow on the photothermal signal was studied in [95]. In a quartz flow cell (diameter 0.5 mm) using an acidic solution of potassium dichromate (1 mmol/L), it was found that at a flow rate of 1.6 mL/min, the thermal lens signal decreased by 5% (at a modulator frequency of 1.25 Hz). An increase in flow resulted in a decrease in signal. A signal-to-noise ratio higher than 5 was obtained up to a flow rate of 5.2 mL/min [95]. With pulsed excitation, the signal magnitude does not depend on the flow velocity [99].

Another source of convection effects may be radiation power. Convection increases with the excitation power [100]. Hence, as a means of solving the problem, the excitation power can be reduced. Thermal conductivity and diffusion coefficient of liquids depend on the effect of molecular diffusion. With increasing temperature, the chaotic nature of molecular movements increases. This prevents heat transfer, which reduces the thermal conductivity of a liquid (with the exception of water). Thus, with increasing radiation power, changes in the characteristic time and thermal diffusivity can be observed, and as noted in [101], the constancy of thermal diffusivity and thermal conductivity indicates the absence of convection and the interfering influence of molecular diffusion on the results of photothermal measurements. Strong convective flow can be observed visually using a magnifying lens in the presence of suspended particles [102]. This leads to the issue of dispersed systems, which will be discussed further. Reducing the excitation beam waist (short focal-length optical focusing lenses) can be a way to reduce the effect of flux on photothermal measurements. Such a reduction will make it possible to reduce the characteristic time of the sample, and thereby use higher modulation frequencies [78].

### 3.5. Ludwig–Soret Effect

The Ludwig–Soret or Soret effect, thermal concentration diffusion, contributes to finding the characteristic time and thermal lens signal. In pure solvents with low optical absorbance, the Soret effect does not appear. On the contrary, in most binary mixtures and finely dispersed systems and micellar solutions, the Soret effect manifests itself [75,103].

**Figure 4:**
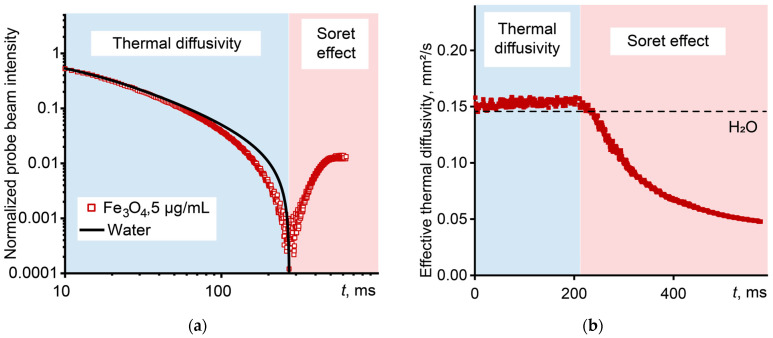
Photothermal measurements of a dispersion of magnetite nanoparticles (5 µg/mL, size 10 nm): (**a**) normalized (in the range of 1–0) transient curve in logarithmic coordinates and (**b**) effective thermal diffusivity.

Figure 4a shows the transient curve of the development of a thermal lens for a dispersion of magnetite nanoparticles in water (5 mg/mL), in which there is first a decrease in intensity and the achievement of a stationary state at 250–270 ms, after which the intensity of the probe beam increases. This anomalous thermal lens signal is due to the formation of a thermally induced concentration gradient leading to a new focal length [104]. In this case, the effective thermal diffusivity of the system also changes (Figure 4b). During the first 250 ms, oscillations around the equilibrium value are observed, after which the effective thermal diffusivity decreases. As the results of [44] showed without taking into account the Soret effect, the error in finding the actual value of thermal diffusivity can be more than 100%.

The influence of concentration diffusion was studied in detail in [103,105,106]. The effect of the concentration gradient depends on the excitation duration and the concentration of the nanophase (or the solvent ratio in mixtures). Working with short irradiation times prevents the effect of mass diffusion; however, if a high modulator frequency is used, concentration diffusion can accumulate due to incomplete relaxation of the thermal lens at the moment the shutter is closed [105]. As noted in [105], when using a modulation frequency of 10 Hz for aqueous solutions of surfactants, an increase in the signal with time was observed. An increase in concentration (or ratio of solvents in mixtures) also affects the effect of mass diffusion. It has been found experimentally that for water–alcohol mixtures (ethanol and propanol), the error caused by the concentration gradient can be up to 25% of the pure thermal lens signal when the volume fraction of the solvent is in the range of 0.05–0.25 [103].

Shen–Snook’s model does not consider thermophoresis. For this reason, to find the true thermal diffusivity, an approximation is carried out to the initial part of the curve, usually up to 400 ms, during which mass diffusion does not act, and the change in the intensity of the probe beam is determined only by the temperature-dependent gradient of the refractive index [103]. The reduction in the probe beam intensity caused by the Soret effect can lead to erroneous results, especially when measurements are carried out in a steady state for quantitative analysis purposes [104].

Another way to reduce systematic error is to use adapted approaches that take into account the Soret effect. Taking into account the thermally induced concentration gradient makes it possible to reduce the error in photothermal measurements of below 1% [107]. However, this approach is more complex and cumbersome to use.

### 3.6. Effect of Solvent

The solvent makes an individual contribution to the measurement error of the thermal lens signal and thermal diffusivity. As previously established, the nature of the solvent affects the reproducibility of thermal lens measurements [108] and the sensitivity of the method [6]. High sensitivity is achieved when using solvents with a high temperature coefficient of refractive index and low thermal conductivity (organic solvents). In particular, the temperature coefficient of the refractive index can be varied in a broad range by changing the solvent composition [6]. Table 2 presents the results of measurements of pure solvents, all other conditions being equal.

Water, as the most common solvent in photothermal practice, requires special consideration. At a temperature of 4 °C, the temperature coefficient of the refractive index of water is zero, which reduces the sensitivity of the TLS to zero [109]. However, as temperature increases, the thermal lens sensitivity increases. Sensitivity can also be increased by adding electrolytes (discussed below) or using mixtures of water with other solvents [70,109]. Water strongly absorbs light at all wavelengths in the UV/vis range. Water absorption has been reported to reach a minimum between 400 and 500 nm [110,111,112]. According to photoacoustic studies, the absorption of water changes slowly in the range of 400–570 nm, with values ranging from 2 to 6·10^–4^ cm^–1^ [113]. At ca. 570 nm, the absorption increases sharply, reaching 2.5 × 10^–3^ cm^–1^ at 600 nm. In addition, the background noise in water is relatively low, which makes water an acceptable solvent for TLS [6].

As noted in [102], the solvent with the least noise in the absence of a dye is carbon tetrachloride. In this case, the values of the thermal lens signal and noise increase in the row: water < methanol, ethanol < n-butanol < n-pentanol, which is consistent with the expected increase in the molar absorptivity of solvents [102]. It is recommended to use HPLC-grade solvents [102].

**Figure 5:**
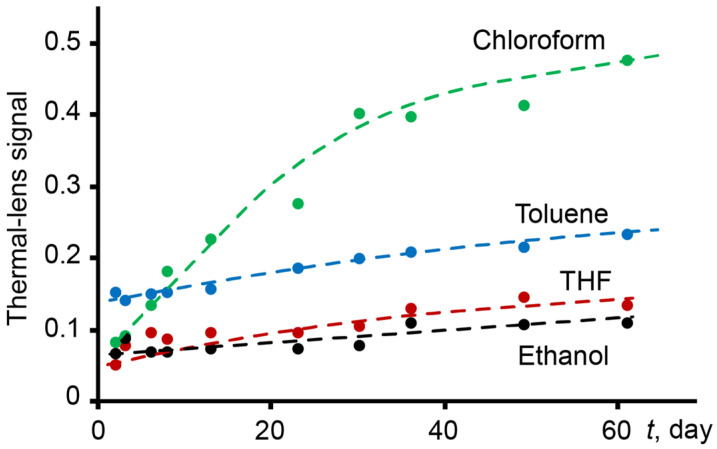
Thermal lens signal for various solvents, measured under other equal conditions (*p* = 62 mW, *n* = 3).

TLS is capable of recording subtle physicochemical changes occurring in samples and it is necessary to strictly monitor the purity of solvents. Repeated and prolonged analysis of the same samples leads to contamination. In this case, a change in the thermal lens signal is observed. In Figure 5, the change in the thermal lens signal for various solvents over time is presented.

### 3.7. Electrolytes

The presence of electrolytes in the test solution affects the thermal lens signal [114,115]. Due to the high sensitivity of TLS, differences due to the presence of salts are observed between tap, distilled, and deionized water, as shown above. The presence of salts enhances the photothermal signal. This is mainly due to changes in the thermo-optical parameters of the solution (refractive index and d*n*/d*T* [116]). An increase in electrolyte concentration most often causes a decrease in thermal conductivity. By removing water molecules from the hydration spheres of ions, the number of hydrogen bonds in water decreases and fewer complexes are available for thermal conduction [116]. In [85], the authors observed a signal enhancement of 45 and 50% when adding 1.35 mol/L KI to water in measurements under continuous and pulsed excitation, respectively.

**Figure 6:**
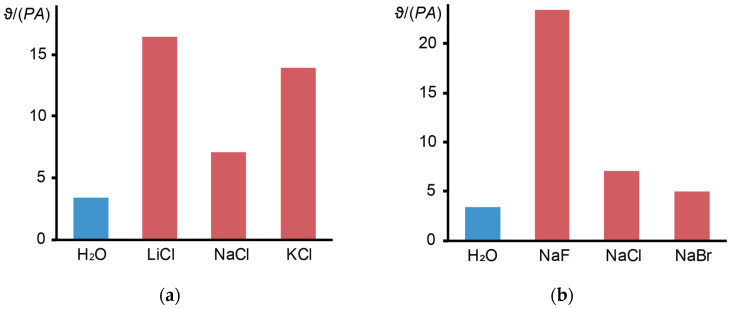
Effects of (**a**) cations and (**b**) anions with a concentration of 1 mol/L on the normalized thermal lens signal for water.

Figure 6 shows the results of measuring the thermal lens signal for various electrolyte solutions. It was found that the smallest sizes of the cation and anion (Li^+^ and F^–^, respectively) have the greatest influence on the thermal lens signal. At the same time, thermal diffusivity for all solutions was within the range of 0.145 ± 0.003 mm^2^/s, which is in good agreement with the theoretical value.

Thus, the addition of electrolytes, on the one hand, helps to increase the sensitivity of thermal lens measurements, but on the other hand, it may affect the correctness of the results of photothermal measurements [1,109,115,117] and, thus, conclusions (e.g., when analyzing biological media, sea waters, etc.).

### 3.8. Photometric Analysis of Multiphase Systems

Multicomponent and finely dispersed systems (dispersions of nano- and microparticles, solutions of proteins and macromolecules, fluorescent and photoinduced systems, etc.) occupy a key place among the samples of interest in photothermal spectroscopy. To date, a large number of works have accumulated in the literature on the use of TLS in the analysis of the thermal and optical properties of heterogeneous systems: dispersions of nano and microparticles and composites, solutions of polymers, proteins, macromolecules, etc. [28,118,119,120]. The high sensitivity of TLS makes it possible to detect changes in the thermal diffusivity of the system at low concentration levels of nano and microphases and solve problems in heat engineering and energy [29,120,121,122]. TLS makes it possible to study systems in a wide range of concentrations (from µg/L to g/L) [28,119,120,123,124] and dispersed phase sizes from several nanometers to hundreds of micrometers [53,120,125,126,127,128]. The analysis of such samples involves solving a number of methodological issues and taking into account factors that depend on the specific sample [75,117,129]. Particularly difficult is the lack of general mathematical approaches to describing the results obtained. In addition, studies of systematic and random errors in the analysis of heterogeneous systems of different nature have not been fully carried out.

The presence of a nano- or microphase significantly affects the physicochemical properties of the entire system. Absorption of radiation by the dispersed phase and subsequent dispersion into the environment leads to the occurrence of side thermal and optical effects (light scattering, concentration and thermal diffusion, convection) that affect the accuracy of measurements. Absorption of radiation can lead to photoinduced physical and chemical processes in the nano-/microphase (aggregation or disaggregation, decomposition, oxidation/reduction, etc.). Most of these effects appear during photothermal analysis and affect stationary and time-resolved signals [44]. On the other hand, the presence of a nano-/microphase in the solvent leads to fluctuations and periodic changes in the signal, which affects the precision of the results [54,130]. This may be a consequence of partial light scattering or local inhomogeneity of the thermal field caused by concentration or thermal diffusion. At the same time, the presence of noise and significant changes in steady-state and time-resolved signals can also be a consequence of physicochemical transformations occurring in the sample. This raises the question of processing the results of photothermal measurements and the correctness of the presented findings and conclusions.

In the following subsections, we consider the features of photothermal analysis of dispersed systems with high and low absorbance and systems with photoinduced activity. As an example of a dispersion with high absorbance, aqueous dispersions of graphene oxide [53] and magnetite nanoparticles were used. Silicon oxide dispersion has been used as an example of a low absorbance sample [44]. The features of photothermal analysis of aqueous solutions of bovine serum albumin and polystyrene nanoparticles are considered as low-absorbance systems. As an example of samples exhibiting photoinduced activity, the behavior of the thermal lens signal and thermal diffusivity of a solution of *p*-chlorophenoxy-substituted lutetium phthalocyanine in chloroform and graphene oxide, which exhibits chemical activity under the action of laser radiation, is considered.

#### 3.8.1. Collection and Processing of Photothermal Data in Disperse Systems

Depending on the dynamics of transient curves, dispersed systems can be divided into heat-conducting and heat-insulating. As a rule, the addition a nano-/microphase improves the thermal properties of the sample, which makes most dispersions classified as thermally conductive [131,132]. In rare cases, due to the parameters of nano-/microphases, dispersions exhibit heat-insulating properties [133].

**Figure 7:**
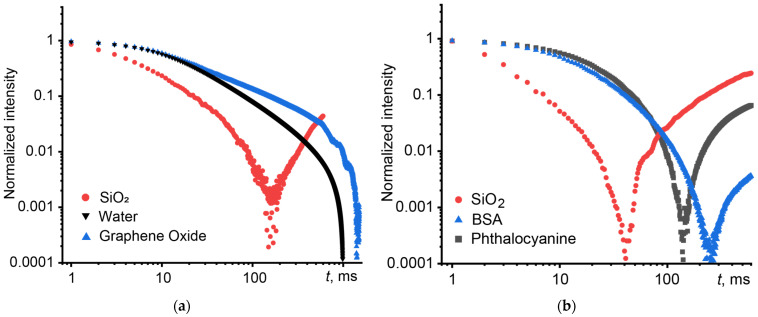
Comparison of normalized transient curves in logarithmic coordinates of complex samples: (**a**) with different absorption and (**b**) with different nature of the nanophase.

For heat-conducting systems, complex behavior of time-resolved and stationary signals is observed. Transient curves of thermal lens development are characterized by rapid achievement of a stationary state and a subsequent increase in intensity due to overheating and/or thermophoresis [44,122,134]. In transient dissipation curves, due to overheating, an extremum is observed, followed by a decrease in intensity and the achievement of an equilibrium state. This behavior was observed for a silicon oxide dispersion (Figure 7a, red line). On the contrary, the graphene oxide dispersion demonstrated thermal insulating properties. This manifests itself in the heating and dissipation transient curves as a longer time to reach steady-state or equilibrium states (Figure 7a, blue line) in comparison with a pure solvent (in both cases it is water, Figure 7a,b, black line). In [44], it was proposed to use the form of transient curves normalized to 1–0 on a logarithmic scale. This form makes it possible to identify and separate the processes of thermal diffusivity and thermophoresis with high accuracy.

Figure 7b shows transient curves for a dispersion of silicon oxide and bovine serum albumin (which do not exhibit significant photochemical properties), as well as a phthalocyanine solution that exhibits photochemical activity. In all cases, a similar shape of the time-resolved signal is observed: an ascending section is observed in the transient curves. In the case of systems with photoinduced activity and photochemical reactions, laser excitation leads to a change in the concentration of absorbing substances in the irradiated volume. Similar photothermal results for different classes can lead to incorrect conclusions in the absence of knowledge of the physicochemical properties of the samples. Here, the question arises of the correctness of photothermal analysis of finely dispersed and multicomponent systems with photochemical activity, the choice of measurement parameters and processing of results.

Shen–Snook’s [65] and Sheldon–Gordon’s [66] models do not take into account the above effects. As shown in [44], using Shen–Snook’s model to find thermal diffusivity without taking into account thermophoresis for an aqueous dispersion of silicon oxide (where it is present) leads to errors of more than 100%. Adaptations of the model proposed in the literature for the analysis of samples with thermal effects and systems with photoinduced activity, as a rule, lead to high accuracy of the results of photothermal measurements; however, they complicate the calculations and require additional measurements (reaction constants, viscosity, etc.) [24], which are not always possible.

In most cases, to solve the problem of measuring the thermal diffusivity of dispersed systems using the Shen–Snook’s model, the characteristic time *t*_c_ and thermal lens signal *ϑ* are selected so that the model and experimental transient curves coincide [128]. In this case, the initial section of the transient curve is used (the first 100–150 ms), i.e., prior the appearance of thermophoresis [135,136].

For a system with photoinduced activity, the thermal diffusivity is determined using similar approaches [137]. However, there is an additional complexity associated with both the choice of the duration of one photothermal measurement cycle and the number of averaging cycles of one measurement. Laser radiation initiates a photochemical process, as a result the concentration of the photoactive component decreases (due to decay, photoinduced disaggregation or agglomeration, aggregation, etc.). This leads to a change in the thermal lens signal with an increase in the number of measurement cycles [53,135]. Hence, the choice of cycle duration and their number depend not only on the required accuracy, but also on the properties of the photochemical system, and it is necessary to take into account the optical properties of the sample and the irradiation power.

To take into account the absorbance of the sample and the power of the excitation beam, the use of a normalized thermal lens signal *ϑ*/(*PA*) was proposed [53]. In the region of low values of *ϑ*/(*PA*), the random error increases, and in the region of high values, the systematic error increases. However, for graphene oxide with a fraction of 1–3.5 kDa, no changes in absorbance are observed (absorbance is below 0.035). This supports the idea that the influence of the morphology and physicochemical properties of the nanophase is more complex and is not limited solely to concentration and absorbance.

**Figure 8:**
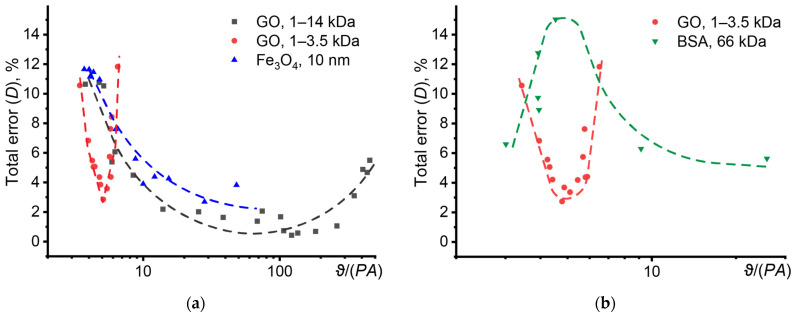
The influence of the normalized thermal lens signal on the error in finding the thermal diffusivity of dispersed systems: (**a**) dispersions of graphene oxide (GO) and magnetite nanoparticles, (**b**) dispersions of graphene oxide and bovine serum albumin (*n* = 3).

To confirm this hypothesis, the analysis of an aqueous solution of bovine serum albumin (Figure 8b) showed an anomalous behavior of the error of thermal diffusivity of the solution from the normalized thermal lens signal. Provided there are no significant changes in absorbance at the initial stage of growth of the thermal lens signal, the error in finding the thermal diffusivity increases, reaching a maximum value at a concentration of 10 mmol/L, after which the error decreases. This behavior has not previously been discovered for disperse systems and the issue requires a separate, more in-depth study.

#### 3.8.2. Optical Properties

According to Shen–Snook’s model, sample absorbance and excitation power play a key role in the dynamics of thermal lens development [65]. For homogeneous systems, high power and absorbance of the sample can lead to excessive heating, which affects the accuracy of determining the thermal diffusivity [54,138]. For complex multicomponent systems and solutions of photochemically active components, increasing absorbance has a similar effect on the accuracy and precision of photothermal measurements. However, the nature of the dispersed phase and photoinduced agents has a more complex impact on the accuracy of photothermal measurements, which is not limited by absorbance.

For disperse systems, finding thermal and optical parameters becomes more difficult. An increase in the amount of the nano/microphase may lead to nonlinear changes in thermal and optical properties [120,127,139]. In most cases, as shown previously [44,53,134], an increase in concentration leads to an increase in absorbance and an increase in the thermal lens signal. With increasing concentration of the nanophase, the thermal diffusivity of the sample, in most cases, changes nonlinearly [120,139,140,141]. In addition to power and absorbance, physical and chemical parameters of the dispersed phase (particle size [141], shape [125,142], coating, etc.) have a significant impact on the accuracy of thermal diffusivity measurements.

**Figure 9:**
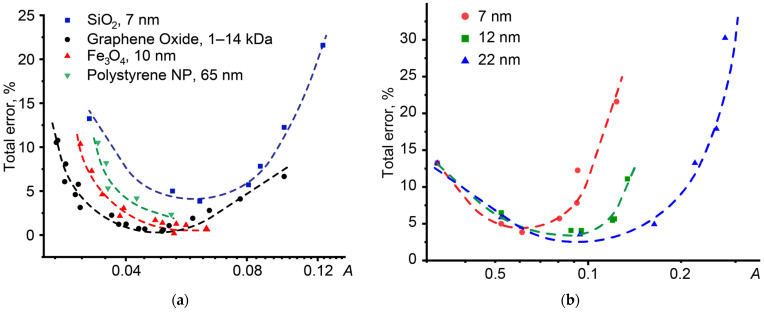
Influence of absorbance on the error in thermal diffusivity for (**a**) aqueous dispersions of polystyrene nanoparticles, magnetite, graphene, and silicon oxides; (**b**) aqueous SiO_2_ dispersions with particle sizes of 7, 12, and 22 nm.

Let us consider the effect of absorbance on the error in determining thermal diffusivity for dispersed systems of various natures (Figure 9a). In all cases, dynamics similar to homogeneous systems are observed: with increasing absorbance, the contribution of the random error decreases, but the contribution of the systematic error increases. However, for dispersions with different absorbances, different ranges of minimum error are observed. For a silicon oxide dispersion (a sample with low absorbance), the range of permissible error (less than 5%) in thermal diffusivity is twofold higher than for a dispersion of graphene oxide (a sample with high absorbance). At the same time, dye-doped polystyrene nanoparticles occupy an intermediate position in terms of absorbance, which is reflected in the error curve.

At the same time, smaller particles have a narrower range of absorbance (than larger particles), in which the thermal diffusivity will be determined with minimal random and systematic error (Figure 9b). Also, with increasing particle size, a shift in the range of the smallest error is observed from the region with low absorbance to the region with higher absorbance. Similar results are demonstrated by graphene oxide dispersions with different molecular weights (Figure 9a).

Data processing plays a major role in the correct interpretation of the results of photothermal measurements of dispersed systems. As found previously [44], for dispersed systems, in addition to the mass concentration, it is necessary to account for the number of particles in solution. This approach takes into account the particle size and provides more reliable results. To identify and subsequently reduce systematic and random errors in measuring the thermal diffusivity of dispersions, it is necessary to measure a series of samples with different absorbances and take into account the morphological, physicochemical, and optical features of the sample.

#### 3.8.3. Thermal Diffusivity

Dispersions of nano-/microparticles are dynamic systems in which physicochemical properties change over time. Solutions of nanoparticles are characterized by processes of aggregation and chemical degradation, which lead to changes in optical and thermal properties [143]. Such changes can be recorded by TLS directly, through the measurement of steady-state and time-resolved signals. However, in a number of cases, the rate and dynamics of spontaneously occurring physical and chemical processes may be insignificant, and changes in thermal and optical properties may be insignificant or commensurate with the magnitude of the measurement error. Thus, the magnitude of the random error can act as an indicator of ongoing changes.

**Figure 10:**
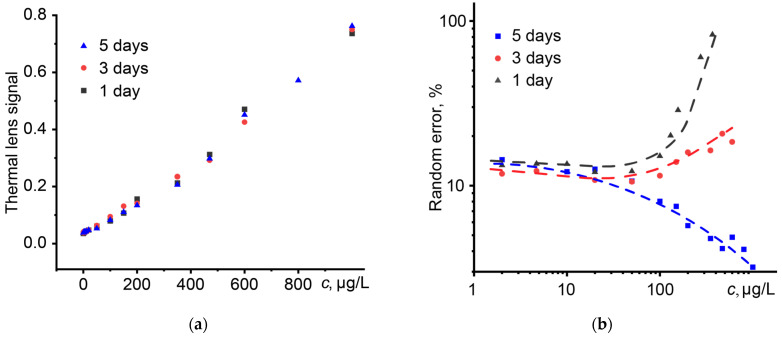
The influence of the concentration of magnetite nanoparticles on (**a**) the thermal lens signal and (**b**) the random error of the signal.

Photothermal analysis of the dispersion of magnetite nanoparticles carried out after 3 and 5 days did not reveal noticeable changes in the thermal diffusivity and thermal lens signal (Figure 10a). At the same time, the random error of the thermal lens signal for different days differs significantly. As is known, nanoparticles with a size of less than 100 nm are prone to aggregation (especially nanoparticles of metals and their oxides) [143,144]. For this reason, their surface modification is carried out with functional polymers, polyelectrolytes, surfactants, etc. [143,144]. In the studied cases, nanoparticles with a size of 10 nm do not have surface modification, and lower random errors of the thermal lens signal may be associated with the aggregation of nanoparticles and the formation of larger structures.

**Figure 11:**
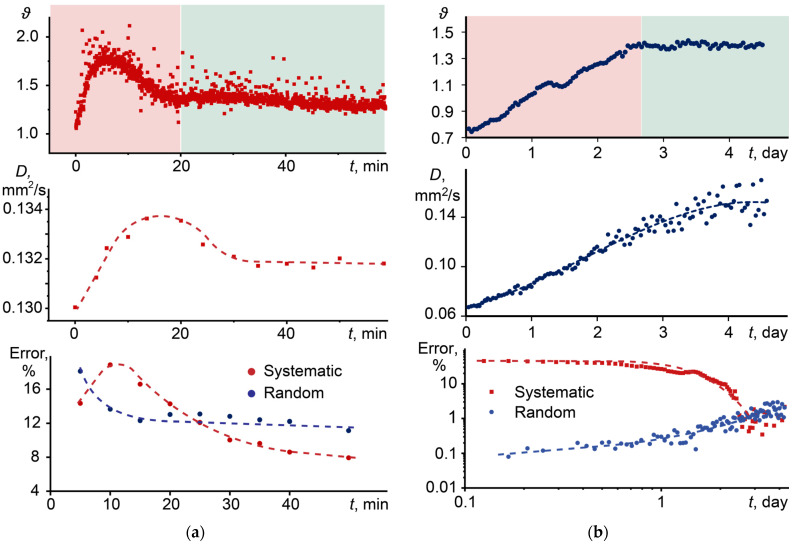
Behavior of the thermal lens signal, thermal diffusivity, and error in the thermal diffusivity for two samples exhibiting photoinduced activity: (**a**) *p*-chlorophenoxy-substituted lutetium phthalocyanine in chloroform and (**b**) an aqueous dispersion of graphene oxide.

Similar results were obtained for samples with photoinduced activity. In such systems, due to laser irradiation, a change in the chromophore concentration occurs, which in some cases leads to a change in physical and thermal properties [24,27,136,137,145,146]. Figure 11a,b shows changes in the thermal lens signal, thermal diffusivity, and the error in thermal diffusivity for a dispersion of graphene oxide and a solution of *p*-chlorophenoxy-substituted lutetium phthalocyanine, exhibiting photoinduced activity.

In both cases, a change in the thermal lens signal and thermal diffusivity was observed during photothermal analysis. For a solution of phthalocyanine in chloroform (Figure 11a), an initial increase and subsequent decrease in the thermal lens signal is observed, reaching a constant value after 20–25 min, which indicates photobleaching. Thermal diffusivity does not change significantly (no more than 2%). For phthalocyanine, the process of photobleaching is due to the formation of singlet oxygen [147].

Due to excitation irradiation, the intensity of the probe beam is first reduced (due to the process of thermal diffusivity), after which photobleaching suppresses the thermal diffusivity. Eventually, the time-resolved signal reaches a steady state, in which photobleaching and mass diffusion change the concentration of absorbing particles in the excited zone, bringing it closer to the equilibrium value. As the number of irradiation cycles increases, the concentration of absorbing particles decreases, which affects the stationary signal. Similar results were observed for Eosin Y solutions, which were also characterized by photobleaching due to the generation of singlet oxygen [27].

Graphene oxide, as found in [53], decomposes into separate layers under the influence of laser radiation within a few days, as a result of which an increase in the thermal lens signal is observed. The change in thermal diffusivity in this case was significant and indicated partial recovery of the exhaust gas.

Finding the thermal diffusivity and thermal lens signal in these cases is complicated by the continuous change of these values over time. In the case of phthalocyanine, we take the value of the thermal lens signal and thermal diffusivity averaged over the interval from 20 to 50 min as the true values (Figure 11a, green area). In this case, for the first 20–25 min, high systematic and random errors are observed (15% and 12%, respectively). In the case of graphene oxide (Figure 11b), using the value of the thermal lens signal averaged in the range from 2.8 to 4 days as the true value (Figure 11b, green area) showed a significant error in finding the thermal diffusivity when compared with the initial values. It is interesting to note that the random error increases over time. But this may be due to an increase in the number of particles [53]. A spontaneous increase in the signal during photothermal analysis can also be the result of an increase in d*n*/d*T* and/or a decrease in thermal conductivity or heat capacity, an increase in heat release, fluorescence quenching, etc. [117]. In each case, there may be several reasons, and it is necessary to consider the individual parameters of each system, which is beyond the scope of this paper.

**Figure 12:**
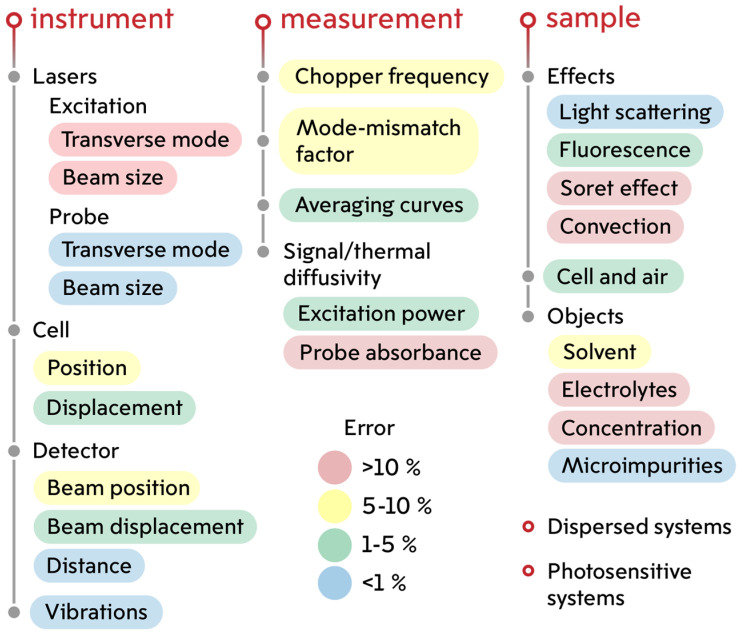
Main factors influencing the accuracy of photothermal measurements.

Thus, photothermal analysis with high accuracy and precision involves taking into account many factors and individual parameters of the sample in each individual case. In addition to instrumental factors and factors associated with measurement parameters, the sources of systematic and random errors are the samples of analysis themselves. As demonstrated by the analysis results presented in this work, light scattering and fluorescence, the presence of electrolytes, and solvent parameters influence the accuracy and precision of thermal diffusivity and photothermal signal measurements. Figure 12 shows a summary chart of the influence of the main factors on the accuracy and precision of thermal lens measurements, taking into account the results presented previously [52,54].

For finely dispersed solutions and systems with photoinduced activity, photothermal analysis, in addition to thermal and optical effects (convection and thermal diffusion, light scattering, etc.), is complicated by the influence of side physical and chemical processes, which, as a rule, have their own nature and influence on the accuracy and precision in each case. Nonlinear changes in the photothermal signal and thermal diffusivity for dispersed solutions and photoinduced systems require taking into account the morphological, chemical and optical features of samples. At the same time, photothermal analysis of complex systems cannot be limited only to the influence of physicochemical parameters of the sample on the optical and thermal parameters.

The value of the relative standard deviation for the thermal lens signal and thermal diffusivity, as well as the thermal lens signal normalized to power and absorbance, are not only an indicator of random and systematic errors, but act as an analytical signal of changes occurring in the system. In this regard, it is necessary to carefully approach the selection of measurement parameters, and also pay attention to identifying systematic and random errors, which are not only an analytical signal of the correctness of measurements but can serve as an indicator of ongoing physicochemical processes.

## 4. Conclusions

Thermal lens spectrometry is a state-of-the-art tool for the physicochemical analysis of samples of various natures. Obtaining photothermal results with high accuracy and precision depends on identifying and taking into account accuracy factors, which are difficult for a multi-signal method such as TLS. This study examined the accuracy factors associated with the sample of analysis and examined the main sources of systematic and random error associated with thermal and optical effects.

The highest influence on the accuracy and precision of measuring thermal and optical properties, among the factors associated with the sample of analysis, is played by thermal diffusion and convection, as well as absorbance and the presence of electrolytes. At the same time, contaminants and light scattering do not affect the accuracy and precision of thermal diffusivity measurements.

The features of photothermal analysis of finely dispersed solutions and systems with photoinduced activity are considered separately. The main issues related to the features of measuring finely dispersed solutions are considered. the need to take into account the influence of self-absorption, the size and mass of the nanophase, and morphological features has been demonstrated. Methods for processing the initial photothermal data play a significant role in obtaining results with high accuracy and precision. As shown in this study, the registration of similar photothermal data for different samples may have a different nature of the changes occurring in the sample. Recommendations for the analysis, processing, and presentation of photothermal measurement results are presented. This study completes the consideration of general issues of the trueness of photothermal measurements of the thermal and optical parameters of liquid samples. Further research should be carried out at a deeper level and the factors of correctness and precision of photothermal analysis should be considered for individual objects with individual characteristics.

The results obtained in this work are relevant for the development and application of photothermal spectroscopy of multicomponent objects. The factors of accuracy and precision associated with the analysis of complex systems (light-absorbing solutions of nanoparticles, proteins, macromolecules, etc.) have not been previously considered in full and generalized. The obtained results not only establish the limits of applicability of TLS in the analysis of disperse systems, but also form the basis for new approaches to assessing the quantitative and qualitative composition of a sample. A new view on the interpretation of the results of thermal lens measurements is proposed, which will open up additional possibilities of photothermal spectroscopy in analytical and applied chemistry.

Despite the found limitations of the method in the analysis of dispersed samples, the results obtained in this study open up additional opportunities in the application of photothermal spectroscopy in the qualitative and quantitative analysis of objects of complex composition. The prospects may be related to TLS of biological objects and living systems, in the analysis of photochemical reactions, fine molecular and disperse processes (solvation, aggregation, disintegration, etc.) with high accuracy.

## Figures and Tables

**Table 1:** Mean thermal lens signals for a quartz cell (path length, 10.00 mm) and air (at 25 °C) at different excitation laser powers.

Sample	Excitation Power, mW	
	200.0	300.0
air	0.00006	0.00022
quartz cell (10.00 mm)	0.00030	0.00124

**Table 2:** Thermal diffusivity and thermal lens signal of solvents measured under equal conditions (*n* = 4, *p* = 300 mW).

Solvent	Thermal Diffusivity, mm^2^/s		Thermal Lens Signal
	Experiment	Theory [70]	
Water (Milli-Q)	0.147 ± 0.005	0.145	0.036 ± 0.003
Water (distilled)	0.149 ± 0.009		0.038 ± 0.003
Water (central water supply)	0.15 ± 0.01		0.046 ± 0.004
Ethanol	0.088 ± 0.002	0.089	0.38 ± 0.02
Chloroform	0.085 ± 0.003	0.083	0.16 ± 0.06
Toluene	0.090 ± 0.008	0.091	1.1 ± 0.3
Acetonitrile	0.115 ± 0.008	0.111	1.120

## Data Availability

The original contributions presented in the study are included in the article/Supplementary Materials, further inquiries can be directed to the corresponding author.

## Supplementary Materials

The following supporting information can be downloaded at: https://www.mdpi.com/article/10.3390/nano14191586/s1, Table S1: Parameters of dispersions of silicon oxide nanoparticles; Table S2: Parameters of dispersions of polystyrene nanoparticles; Table S3: Thermal lens measurement parameters.

## References

[1] Bialkowski S.E., Astrath N.G.C., Proskurnin M.A. (2019). Photothermal Spectroscopy Methods.

[2] Jansen K.L., Harris J.M. (1985). Thermal lens measurements of optical computation of the laser beam spot size. Anal. Chem..

[3] Jansen K.L., Harris J.M. (1985). Double-beam thermal lens spectrometry. Anal. Chem..

[4] Dovichi N.J., Harris J.M. (1981). Time-resolved thermal lens calorimetry. Anal. Chem..

[5] Hannachi R. (2021). Photothermal lens spectrometry: Experimental optimization and direct quantification of permanganate in water. Sens. Actuat. B.

[6] Ramis-Ramos G. (1993). Analytical characteristics, applications and perspectives in thermal lens spectrometry. Anal. Chim. Acta.

[7] Soto C., Saavedra R., Toral M.I., Nacaratte F., Poza C. (2016). Preliminary studies for ciclopirox olamine determination by thermal lens spectrophotometry. Microchem. J..

[8] Cruz R.A., Filadelpho M.C., Castro M.P., Andrade A.A., Souza C.M., Catunda T. (2011). Very low optical absorptions and analyte concentrations in water measured by Optimized Thermal Lens Spectrometry. Talanta.

[9] Kazemi E., Haji Shabani A.M., Dadfarnia S., Abbasi A., Rashidian Vaziri M.R., Behjat A. (2016). Development of a novel mixed hemimicelles dispersive micro solid phase extraction using 1-hexadecyl-3-methylimidazolium bromide coated magnetic graphene for the separation and preconcentration of fluoxetine in different matrices before its determination by fiber optic linear array spectrophotometry and mode-mismatched thermal lens spectroscopy. Anal. Chim. Acta.

[10] Proskurnin M.A., Kononets M.Y. (2004). Modern analytical thermooptical spectroscopy. Russ. Chem. Rev..

[11] Martelanc M., Ziberna L., Passamonti S., Franko M. (2016). Application of high-performance liquid chromatography combined with ultra-sensitive thermal lens spectrometric detection for simultaneous biliverdin and bilirubin assessment at trace levels in human serum. Talanta.

[12] Franko M. (2008). Thermal Lens Spectrometric Detection in Flow Injection Analysis and Separation Techniques. Appl. Spectrosc. Rev..

[13] Šikovec M., Novič M., Franko M. (1996). Application of thermal lens spectrometric detection to the determination of heavy metals by ion chromatography. J. Chromatogr. A.

[14] Hibara A., Fukuyama M., Chung M., Priest C., Proskurnin M.A. (2016). Interfacial Phenomena and Fluid Control in Micro/Nanofluidics. Anal. Sci..

[15] Kurian A., Bindhu C.V., Nampoori V.P.N. (2015). Kinetic Studies of Chemical Reaction using Laser Induced Thermal Lens Technique. J. Opt..

[16] Proskurnin M.A., Chernysh V.V., Pakhomova S.V., Kononets M.Y., Sheshenev A.A. (2002). Investigation of the reaction of copper(I) with 2,9-dimethyl-1,10-phenanthroline at trace level by thermal lensing. Talanta.

[17] Pakhomova S.V., Proskurnin M.A., Chernysh V.V., Kononets M.Y., Ivanova E.K. (2001). Determination of stability constants of copper(I) chelates with 1,10-phenanthroline by thermal lensing. J. Anal. Chem..

[18] Nedosekin D.A., Proskurnin M.A., Kononets M.Y. (2005). Model for continuous-wave laser-induced thermal lens spectrometry of optically transparent surface-absorbing solids. Appl. Opt..

[19] Li S.-H., He H.-B., Shan Y.-G., Li D.-W., Zhao Y.-A., Fan Z.-X. (2010). Enhanced surface thermal lensing for absorption evaluation and defect identification of optical films. Appl. Opt..

[20] Bourgoin J.-P., Allogho G.-G., Haché A. (2010). Thermal conduction in thin films measured by optical surface thermal lensing. J. Appl. Phys..

[21] Seidman K., Payne A. (1998). The Determination of the Heat Capacities of Liquids with Time Resolved Thermal Lens Calorimetry: A More Accurate Procedure. J. Chem. Educ..

[22] Mohebbifar M.R. (2020). Experimental comparison of methods based on falling and rising signal regions for thermal diffusivity measurement by pulsed dual-beam thermal lens spectroscopy. Measurement.

[23] Boudebs G., Zinoune J.B., Cassagne C., Chis M. (2023). Thermal lens Z-scan measurements: Theoretical and experimental uncertainties for low and high fluorescence quantum yields. Appl. Opt..

[24] Astrath N.G., Astrath F.B., Shen J., Zhou J., Michaelian K.H., Fairbridge C., Malacarne L.C., Pedreira P.R., Medina A.N., Baesso M.L. (2009). Thermal-lens study of photochemical reaction kinetics. Opt. Lett..

[25] Brennetot R., Georges J. (1999). Investigation of non-linear absorption effects in pulsed-laser thermal lens spectrometry of dye solutions. Spectrochim. Acta Part A Mol. Biomol. Spectrosc..

[26] Liu M., Ding C., Wang J. (2016). Modeling of thermal conductivity of nanofluids considering aggregation and interfacial thermal resistance. RSC Adv..

[27] Herculano L.S., Malacarne L.C., Zanuto V.S., Lukasievicz G.V., Capeloto O.A., Astrath N.G. (2013). Investigation of the photobleaching process of eosin Y in aqueous solution by thermal lens spectroscopy. J. Phys. Chem. B.

[28] Sindhu Swapna M.N., Raj V., Cabrera H., Sankararaman S.I. (2021). Thermal Lensing of Multi-walled Carbon Nanotube Solutions as Heat Transfer Nanofluids. ACS Appl. Nano Mater..

[29] Swapna M.S., Raj V., Sankararaman S. (2019). Allotropic transformation instigated thermal diffusivity of soot nanofluid: Thermal lens study. Phys. Fluids.

[30] Usoltseva L.O., Volkov D.S., Avramenko N.V., Korobov M.V., Proskurnin M.A. (2018). Nanodiamond aqueous dispersions as potential nanofluids: The determination of properties by thermal lensing and other techniques. Nanosyst. Phys. Chem. Math..

[31] Lopes C.S., Lenart V.M., Turchiello R.F., Gómez S.L. (2018). Determination of the Thermal Diffusivity of Plasmonic Nanofluids Containing PVP-Coated Ag Nanoparticles Using Mode-Mismatched Dual-Beam Thermal Lens Technique. Adv. Condens. Matter Phys..

[32] Georges J. (1999). Advantages and limitations of thermal lens spectrometry over conventional spectrophotometry for absorbance measurements. Talanta.

[33] Thorne J.B., Bobbitt D.R. (1993). Comparison of Beer’s Law and Thermal Lens Techniques for Absorption Measurements under Conditions of High Scattering Backgrounds. Appl. Spectrosc..

[34] Proskurnin M.A., Volkov D.S., Gor’kova T.A., Bendrysheva S.N., Smirnova A.P., Nedosekin D.A. (2015). Advances in thermal lens spectrometry. J. Anal. Chem..

[35] Proskurnin M.A., Khabibullin V.R., Usoltseva L.O., Vyrko E.A., Mikheev I.V., Volkov D.S. (2022). Photothermal and optoacoustic spectroscopy: State of the art and prospects. Phys. Uspekhi.

[36] Bauer S., De Reggi A.S. (1996). Pulsed electrothermal technique for measuring the thermal diffusivity of dielectric films on conducting substrates. J. Appl. Phys..

[37] Prado J.I., Calviño U., Lugo L. (2022). Experimental Methodology to Determine Thermal Conductivity of Nanofluids by Using a Commercial Transient Hot-Wire Device. Appl. Sci..

[38] Hu R., Ma A., Wang Y. (2018). Transient hot wire measures thermophysical properties of organic foam thermal insulation materials. Exp. Therm Fluid Sci..

[39] Jaber W., Chapuis P.-O. (2018). Non-idealities in the 3ω method for thermal characterization in the low- and high-frequency regimes. AIP Adv..

[40] Usoltseva L.O., Volkov D.S., Karpushkin E.A., Korobov M.V., Proskurnin M.A. (2021). Thermal Conductivity of Detonation Nanodiamond Hydrogels and Hydrosols by Direct Heat Flux Measurements. Gels.

[41] Souza R.R., Faustino V., Gonçalves I.M., Moita A.S., Bañobre-López M., Lima R. (2022). A Review of the Advances and Challenges in Measuring the Thermal Conductivity of Nanofluids. Nanomaterials.

[42] Usoltseva L.O., Korobov M.V., Proskurnin M.A. (2020). Photothermal spectroscopy: A promising tool for nanofluids. J. Appl. Phys..

[43] Qiu L., Ma Y., Wang S., Ouyang Y., Feng Y. (2023). High-accuracy thermophysical property measurement technique based on Labview-3ω method. AIP Adv..

[44] Khabibullin V.R., Usoltseva L.O., Mikheev I.V., Proskurnin M.A. (2023). Thermal Diffusivity of Aqueous Dispersions of Silicon Oxide Nanoparticles by Dual-Beam Thermal Lens Spectrometry. Nanomaterials.

[45] Nunes dos Santos W., Mummery P., Wallwork A. (2005). Thermal diffusivity of polymers by the laser flash technique. Polym. Test..

[46] Venerus D.C., Schieber J.D., Iddir H., Guzman J.D., Broerman A.W. (1999). Measurement of thermal diffusivity in polymer melts using forced Rayleigh light scattering. J. Polym. Sci. Part B Polym. Phys..

[47] Bennett C.A., Patty R.R. (1982). Thermal wave interferometry: A potential application of the photoacoustic effect. Appl. Opt..

[48] Lang S.B. (1989). Technique for the measurement of thermal diffusivity based on the laser intensity modulation method (LIMM). Ferroelectrics.

[49] Habiba U., Hebert R.J. (2023). Powder Bed Thermal Diffusivity Using Laser Flash Three Layer Analysis. Materials.

[50] Philipp A., Eichinger J.F., Aydin R.C., Georgiadis A., Cyron C.J., Retsch M. (2019). The accuracy of laser flash analysis explored by finite element method and numerical fitting. Heat Mass Transf..

[51] Bento A.C., Almond D.P. (1995). The accuracy of thermal wave interferometry for the evaluation of thermophysical properties of plasma-sprayed coatings. Meas. Sci. Technol..

[52] Khabibullin V.R., Usoltseva L.O., Galkina P.A., Galimova V.R., Volkov D.S., Mikheev I.V., Proskurnin M.A. (2023). Measurement Precision and Thermal and Absorption Properties of Nanostructures in Aqueous Solutions by Transient and Steady-State Thermal-Lens Spectrometry. Physchem.

[53] Khabibullin V.R., Ratova D.V., Stolbov D.N., Mikheev I.V., Proskurnin M.A. (2023). The Thermophysical and Physicochemical Properties of the Aqueous Dispersion of Graphene Oxide Dual-Beam Thermal Lens Spectrometry. Nanomaterials.

[54] Khabibullin V.R., Franko M., Proskurnin M.A. (2023). Accuracy of Measurements of Thermophysical Parameters by Dual-Beam Thermal-Lens Spectrometry. Nanomaterials.

[55] Matsumoto I., Sekiya R., Haino T. (2019). A protocol for size separation of nanographenes. RSC Adv..

[56] Shevchenko N., Tomsik E., Laishevkina S., Iakobson O., Pankova G. (2021). Cross-linked polyelectrolyte microspheres: Preparation and new insights into electro-surface properties. Soft Matter.

[57] Shakirova J.R., Shevchenko N.N., Baigildin V.A., Chelushkin P.S., Khlebnikov A.F., Tomashenko O.A., Solomatina A.I., Starova G.L., Tunik S.P. (2019). Eu-Based Phosphorescence Lifetime Polymer Nanothermometer: A Nanoemulsion Polymerization Approach to Eliminate Quenching of Eu Emission in Aqueous Media. ACS Appl. Polym. Mater..

[58] Kazimirova K.O., Shtykov S.N. (2024). Sorption and concentration anionic azo dyes on nanomagnetite modified with cationic polyelectrolytes. Sorbtsionnye I Khromatograficheskie Protsessy.

[59] Egunova O.R., Reshetnikova I.S., Kazimirova K.O., Shtykov S.N. (2020). Magnetic Solid-Phase Extraction and Fluorimetric Determination of Some Fluoroquinolones. J. Anal. Chem..

[60] Dubinina T.V., Tomilova L.G., Zefirov N.S. (2013). Synthesis of phthalocyanines with an extended system of π-electron conjugation. Russ. Chem. Rev..

[61] Dubinina T.V., Paramonova K.V., Trashin S.A., Borisova N.E., Tomilova L.G., Zefirov N.S. (2014). Novel near-IR absorbing phenyl-substituted phthalo- and naphthalocyanine complexes of lanthanide(III): Synthesis and spectral and electrochemical properties. Dalton Trans..

[62] Carter C.A., Harris J.M. (1984). Comparison of models describing the thermal lens effect. Appl. Opt..

[63] Gordon J.P., Leite R.C.C., Moore R.S., Porto S.P.S., Whinnery J.R. (1965). Long-Transient Effects in Lasers with Inserted Liquid Samples. J. Appl. Phys..

[64] Hu C., Whinnery J.R. (1973). New thermooptical measurement method and a comparison with other methods. Appl. Opt..

[65] Shen J., Lowe R.D., Snook R.D. (1992). A model for cw laser induced mode-mismatched dual-beam thermal lens spectrometry. Chem. Phys..

[66] Sheldon S.J., Knight L.V., Thorne J.M. (1982). Laser-induced thermal lens effect: A new theoretical model. Appl. Opt..

[67] Vargas-Vargas A.D., Mejorada-Sánchez J.L., Castellanos-Durán F.R., Vargas E., Isidro-Ojeda M.A., Cedeño E., Rojas-Trigos J.B., Calderón A., Marín E. (2023). Dual beam transient thermal lens spectroscopy with high repetition pulsed IR-Laser Excitation: Photothermal and fluorescence quantum yields determination. Infrared Phys. Technol..

[68] Proskurnin M.A., Usoltseva L.O., Volkov D.S., Nedosekin D.A., Korobov M.V., Zharov V.P. (2021). Photothermal and Heat-Transfer Properties of Aqueous Detonation Nanodiamonds by Photothermal Microscopy and Transient Spectroscopy. J. Phys. Chem. C.

[69] Mikheev I.V., Usoltseva L.O., Ivshukov D.A., Volkov D.S., Korobov M.V., Proskurnin M.A. (2016). Approach to the Assessment of Size-Dependent Thermal Properties of Disperse Solutions: Time-Resolved Photothermal Lensing of Aqueous Pristine Fullerenes C60and C70. J. Phys. Chem. C.

[70] Dovichi N.J., Bialkowski S.E. (1987). Thermo-Optical Spectrophotometries in Analytical Chemistry. C R C Crit. Rev. Anal. Chem..

[71] Franko M., Tran C.D. (1996). Analytical thermal lens instrumentation. Rev. Sci. Instrum..

[72] Soyeh I., Hannachi R., Sammouda H. (2023). Investigation of the quartz cuvette surface contamination used for permanganate quantification in tap water by photothermal lens spectrometry. Appl. Phys. B.

[73] Proskurnin M.A., Bendrysheva S.N., Ragozina N., Heissler S., Faubel W., Pyell U. (2005). Optimization of instrumental parameters of a near-field thermal-lens detector for capillary electrophoresis. Appl. Spectrosc..

[74] Zidan M.D., Allahham A., Ghanem A., Mousa N., Abdallah B., Al Salman A. (2024). Thermal lens investigation of the CdSe quantum dots using dual beam z-scan technique. Results Opt..

[75] Arnaud N., Georges J. (2001). Thermal lens spectrometry in aqueous solutions of Brij 35: Investigation of micelle effects on the time-resolved and steady-state signals. Spectrochim. Acta A Mol. Biomol. Spectrosc..

[76] Escalona R. (2008). Study of a convective field induced by thermal lensing using interferometry. Opt. Commun..

[77] Vyrko E., Volkov D.S., Proskurnin M.A. (2018). Numerical Simulation of Photothermal Lens Spectrometry Models Relevant for Analytical Chemistry. Int. J. Thermophys..

[78] Carter C.A., Harris J.M. (1984). Thermal lens absorption measurements on small volume samples. Anal. Chem..

[79] Dada O.O., Jorgensen M.R., Bialkowski S.E. (2007). Continuous laser-excited photothermal spectrometry of CdS_x_Se_1-x_ doped glasses. Appl. Spectrosc..

[80] Joshi P.R., Dada O.O., Bialkowski S.E. (2010). Pulsed laser excited photothermal lens spectrometry of cadmium sulfoselenide doped silica glasses. J. Phys. Conf. Ser..

[81] Power J.F., Langford C.H. (1988). Optical absorbance of dissolved organic matter in natural water studies using the thermal lens effect. Anal. Chem..

[82] Yasa Z.A., Jackson W.B., Amer N.M. (1982). Photothermal spectroscopy of scattering media. Appl. Opt..

[83] Šikovec M., Cruz F.G., Franko M., Katz S.A. (1996). Determination of Hexavalent Chromium in Extracts of CCA-Treated Building Timbers By Thermal Lens Spectrometry: A Comparison to Spectrophotometry and Atomic Absorption Spectrometry. Spectrosc. Lett..

[84] Marcano A., Basaldua I., Villette A., Edziah R., Liu J., Ziane O., Melikechi N. (2013). Photothermal lens spectrometry measurements in highly turbid media. Appl. Spectrosc..

[85] Georges J., Mermet J.M. (1994). Measurement of thermal energy recovery using thermal lens spectrometry in fluorescence quenching experiments. Spectrochim. Acta Part A Mol. Spectrosc..

[86] Fischer M., Georges J. (1996). Fluorescence quantum yield of rhodamine 6G in ethanol as a function of concentration using thermal lens spectrometry. Chem. Phys. Lett..

[87] Fischer M., Georges J. (1997). Use of thermal lens spectrometry for the investigation of dimerization equilibria of rhodamine 6G in water and aqueous micellar solutions. Spectrochim. Acta Part A Mol. Biomol. Spectrosc..

[88] Dy E., Gu C., Shen J., Qu W., Xie Z., Wang X., Baesso M.L., Astrath N.G.C. (2022). Sensitivity enhancement of thermal lens spectrometry. J. Appl. Phys..

[89] Malacarne L.C., Savi E.L., Baesso M.L., Lenzi E.K., Astrath N.G.C. (2014). Role of Photophysics Processes in Thermal Lens Spectroscopy of Fluids: A Theoretical Study. J. Phys. Chem. A.

[90] Georges J., Arnaud N., Parise L. (1996). Limitations arising from optical saturation in fluorescence and thermal lens spectrometries using pulsed laser excitation: Application to the determination of the fluorescence quantum yield of rhodamine 6G. Appl. Spectrosc..

[91] Shemeena Basheer N., Rajesh Kumar B., Kurian A., George S.D. (2013). Thermal lens probing of distant dependent fluorescence quenching of Rhodamine 6G by silver nanoparticles. J. Lumin..

[92] Estupiñán-López C., Dominguez C.T., Filho P.E.C., Santos B.S., Fontes A., de Araujo R.E. (2016). A pH dependence study of CdTe quantum dots fluorescence quantum yields using eclipsing thermal lens spectroscopy. J. Lumin..

[93] Fischer M., Georges J. (1998). Limitations arising in the study of the fluorescence quenching of rhodamine 6G by iodides using cw-laser thermal lens spectrometry. Spectrochim. Acta Part A Mol. Biomol. Spectrosc..

[94] Singhal S., Goswami D. (2020). Unraveling the molecular dependence of femtosecond laser-induced thermal lens spectroscopy in fluids. Analyst.

[95] Amador-Hernandez J., Fernandez-Romero J.M., Ramis-Ramos G., De Castro M.D.L. (1998). Pulse Thermal Lens Spectrometry of β-Carotene in Flow Systems at Atmospheric- and High-Pressure Conditions. Appl. Spectrosc..

[96] Buffett C.E., Morris M.D. (1983). Convective Effects in Thermal Lens Spectroscopy. Appl. Spectrosc..

[97] Dovichi N.J., Harris J.M. (1981). Thermal lens calorimetry for flowing samples. Anal. Chem..

[98] Skogerboe K.J., Yeung E.S. (1986). Single laser thermal lens detector for microbore liquid chromatography based on high-frequency modulation. Anal. Chem..

[99] Nickolaisen S.L., Bialkowski S.E. (1986). Pulsed laser thermal lens spectrophotometry for flowing liquid detection. Anal Chem.

[100] Alfheim J.A., Langford C.H. (1985). Determination of formaldehyde with the thermal lens effect. Anal. Chem..

[101] Mohebbifar M.R. (2021). Study of the effect of temperature on thermophysical properties of ethyl myristate by dual-beam thermal lens technique. Optik.

[102] Martín-Biosca Y., Medina-Hernández M.J., García-Alvarez-Coque M.C., Ramis-Ramos G. (1994). Effect of the nature of the solvent on the limit of detection in thermal lens spectrometry. Anal. Chim. Acta.

[103] Arnaud N., Georges J. (2004). Cw-laser thermal lens spectrometry in binary mixtures of water and organic solvents: Composition dependence of the steady-state and time-resolved signals. Spectrochim. Acta. A Mol. Biomol. Spectrosc..

[104] Georges J., Paris T. (1999). Influence of the Soret effect on the analytical signal in cw-laser thermal lens spectrometry of micellar solutions. Anal. Chim. Acta.

[105] Arnaud N., Georges J. (2001). On the analytical use of the Soret-enhanced thermal lens signal in aqueous solutions. Anal. Chim. Acta.

[106] Arnaud N., Georges J. (2001). Investigation of the thermal lens effect in water-ethanol mixtures: Composition dependence of the refractive index gradient, the enhancement factor and the Soret effect. Spectrochim. Acta. A Mol. Biomol. Spectrosc..

[107] Cabrera H., Sira E., Rahn K., García-Sucre M. (2009). A thermal lens model including the Soret effect. Appl. Phys. Lett..

[108] Proskurnin M.A., Chernysh V.V., Filichkina V.A. (2004). Some Metrological Aspects of the Optimization of Thermal-Lens Procedures. J. Anal. Chem..

[109] Franko M., Tran C.D. (2002). Thermal lens effect in electrolyte and surfactant media. J. Phys. Chem..

[110] Hornig D. (1964). On the Spectrum and Structure of Water and Ionic Solutions. J. Chem. Phys..

[111] Pope R.M., Fry E.S. (1997). Absorption spectrum (380–700 nm) of pure water. 2. Integrating cavity measurements. Appl. Opt..

[112] Sogandares F.M., Fry E.S. (1997). Absorption spectrum (340–640 nm) of pure water. I. Photothermal measurements. Appl. Opt..

[113] Patel C.K.N., Tam A.C. (1979). Optical absorption coefficients of water. Nature.

[114] Phillips C.M., Crouch S.R., Leroi G.E. (1986). Matrix effects in thermal lensing spectrometry: Determination of phosphate in saline solutions. Anal. Chem..

[115] Zhirkov A.A., Nikiforov A.A., Tsar’kov D.S., Volkov D.S., Proskurnin M.A., Zuev B.K. (2012). Effect of electrolytes on the sensitivity of the thermal lens determination. J. Anal. Chem..

[116] Colcombe S.M., Lowe R.D., Snook R.D. (1997). Thermal lens investigation of the temperature dependence of the refractive index of aqueous electrolyte solutions. Anal. Chim. Acta.

[117] Georges J. (2008). Matrix effects in thermal lens spectrometry: Influence of salts, surfactants, polymers and solvent mixtures. Spectrochim. Acta. A Mol. Biomol. Spectrosc..

[118] Simon J., Anugop B., Nampoori V.P.N., Kailasnath M. (2021). Effect of pulsed laser irradiation on the thermal diffusivity of bimetallic Au/Ag nanoparticles. Opt. Laser Technol..

[119] Jiménez-Pérez J.L., López-Gamboa G., Sánchez-Ramírez J.F., Correa-Pacheco Z.N., Netzahual-Lopantzi A., Cruz-Orea A. (2021). Thermal Diffusivity Dependence with Highly Concentrated Graphene Oxide/Water Nanofluids by Mode-Mismatched Dual-Beam Thermal Lens Technique. Int. J. Thermophys..

[120] Nideep T.K., Ramya M., Nampoori V.P.N., Kailasnath M. (2020). The size dependent thermal diffusivity of water soluble CdTe quantum dots using dual beam thermal lens spectroscopy. Phys. E Low-Dimens. Syst. Nanostructures.

[121] Swapna M.N.S., Korte D., Sankararaman S.I. (2023). Solid-Volume-Fraction Retained Tailoring of Thermal Diffusivity of Multiwalled Carbon Nanotube Nanofluid: A Photothermal Investigation. Phys. Status Solidi.

[122] Netzahual-Lopantzi Á., Sánchez-Ramírez J.F., Jiménez-Pérez J.L., Cornejo-Monroy D., López-Gamboa G., Correa-Pacheco Z.N. (2019). Study of the thermal diffusivity of nanofluids containing SiO_2_ decorated with Au nanoparticles by thermal lens spectroscopy. Appl. Phys. A.

[123] Prakash A., Pathrose B.P., Nampoori V.P.N., Radhakrishnan P., Mujeeb A. (2019). Thermal diffusivity of neutral red dye using dual beam thermal lens technique: A comparison on the effects using nano pulsed laser ablated silver and gold nanoparticles. Phys. E Low-Dimens. Syst. Nanostructures.

[124] Luna-Sánchez J.L., Jiménez-Pérez J.L., Carbajal-Valdez R., Lopez-Gamboa G., Pérez-González M., Correa-Pacheco Z.N. (2019). Green synthesis of silver nanoparticles using Jalapeño Chili extract and thermal lens study of acrylic resin nanocomposites. Thermochim. Acta.

[125] Lopez-Munoz G.A., Pescador-Rojas J.A., Ortega-Lopez J., Salazar J.S., Balderas-Lopez J.A. (2012). Thermal diffusivity measurement of spherical gold nanofluids of different sizes/concentrations. Nanoscale Res. Lett..

[126] Mathew R.M., Zachariah E.S., Jose J., Thomas T., John J., Titus T., Unni N.G., Mathew S., Mujeeb A., Thomas V. (2020). Synthesis, characterization and evaluation of tunable thermal diffusivity of phosphorus-doped carbon nanodot. Appl. Phys. A.

[127] Ramya M., Nideep T.K., Nampoori V.P.N., Kailasnath M. (2019). Particle size and concentration effect on thermal diffusivity of water-based ZnO nanofluid using the dual-beam thermal lens technique. Appl. Phys. B.

[128] Jiménez-Pérez J.L., Gutiérrez Fuentes R., Sánchez-Sosa R., Zapata Torres M.G., Correa-Pacheco Z.N., Sánchez Ramírez J.F. (2015). Thermal diffusivity study of nanoparticles and nanorods of titanium dioxide (TiO_2_) and titanium dioxide coated with cadmium sulfide (TiO2CdS). Mater. Sci. Semicond. Process..

[129] González-Araoz M.P., Sánchez-Ramírez J.F., Jiménez-Pérez J.L., Chigo-Anota E., Herrera-Pérez J.L., Mendoza-Álvarez J.G.J.N.S. (2012). Negative thermal diffusivity enhancement in semiconductor nanofluids. Nat. Sci..

[130] Augustine A.K., Mathew S., Girijavallabhan C.P., Radhakrishnan P., Nampoori V.P.N., Kailasnath M. (2014). Size dependent variation of thermal diffusivity of CdSe nanoparticles based nanofluid using laser induced mode-matched thermal lens technique. J. Opt..

[131] Moreira T.A., Moreira D.C., Ribatski G. (2018). Nanofluids for heat transfer applications: A review. J. Braz. Soc. Mech. Sci. Eng..

[132] Tao Q., Zhong F., Deng Y., Wang Y., Su C. (2023). A Review of Nanofluids as Coolants for Thermal Management Systems in Fuel Cell Vehicles. Nanomaterials.

[133] Fernandez I., Valiente R., Ortiz F., Renedo C.J., Ortiz A. (2020). Effect of TiO_2_ and ZnO Nanoparticles on the Performance of Dielectric Nanofluids Based on Vegetable Esters During Their Aging. Nanomaterials.

[134] Herrera-Aquino R., Jiménez-Pérez J.L., Altamirano-Juárez D.C., López-Gamboa G., Correa-Pacheco Z.N., Carbajal-Valdéz R. (2018). Green Synthesis of Silver Nanoparticles Contained in Centrifuged Citrus Oil and Their Thermal Diffusivity Study by Using Thermal Lens Technique. Int. J. Thermophys..

[135] Pedreira P.R.B., Hirsch L.R., Pereira J.R.D., Medina A.N., Bento A.C., Baesso M.L., Rollemberg M.C.E., Franko M. (2004). Observation of laser induced photochemical reaction of Cr(VI) species in water during thermal lens measurements. Chem. Phys. Lett..

[136] Pedreira P.R.B., Hirsch L.R., Pereira J.R.D., Medina A.N., Bento A.C., Baesso M.L., Rollemberg M.C., Franko M., Shen J. (2006). Real-time quantitative investigation of photochemical reaction using thermal lens measurements: Theory and experiment. J. Appl. Phys..

[137] Savi E.L., Malacarne L.C., Baesso M.L., Pintro P.T.M., Croge C., Shen J., Astrath N.G.C. (2015). Investigation into photostability of soybean oils by thermal lens spectroscopy. Spectrochim. Acta. A Mol. Biomol. Spectrosc..

[138] Rodriguez L.G., Iza P., Paz J.L. (2016). Study of dependence between thermal diffusivity and sample concentration measured by means of frequency-resolved thermal lens experiment. J. Nonlinear Opt. Phys. Mater..

[139] Mathew S., Francis F., Joseph S.A., Kala M.S. (2021). Enhanced thermal diffusivity of water based ZnO nanoflower/rGO nanofluid using the dual-beam thermal lens technique. Nano-Struct. Nano-Objects.

[140] Francis F., Anila E.I., Joseph S.A. (2020). Dependence of thermal diffusivity on nanoparticle shape deduced through thermal lens technique taking ZnO nanoparticles and nanorods as inclusions in homogeneous dye solution. Optik.

[141] Lenart V.M., Astrath N.G.C., Turchiello R.F., Goya G.F., Gómez S.L. (2018). Thermal diffusivity of ferrofluids as a function of particle size determined using the mode-mismatched dual-beam thermal lens technique. J. Appl. Phys..

[142] Hari M., Joseph S.A., Mathew S., Nithyaja B., Nampoori V.P.N., Radhakrishnan P. (2013). Thermal diffusivity of nanofluids composed of rod-shaped silver nanoparticles. Int. J. Therm. Sci..

[143] Nguyen M.D., Tran H.-V., Xu S., Lee T.R. (2021). Fe_3_O_4_ Nanoparticles: Structures, Synthesis, Magnetic Properties, Surface Functionalization, and Emerging Applications. Appl. Sci..

[144] Khabibullin V.R., Chetyrkina M.R., Obydennyy S.I., Maksimov S.V., Stepanov G.V., Shtykov S.N. (2023). Study on Doxorubicin Loading on Differently Functionalized Iron Oxide Nanoparticles: Implications for Controlled Drug-Delivery Application. Int. J. Mol. Sci..

[145] Constantino R., Lenzi G.G., Franco M.G., Lenzi E.K., Bento A.C., Astrath N.G.C., Malacarne L.C., Baesso M.L. (2017). Thermal Lens Temperature Scanning technique for evaluation of oxidative stability and time of transesterification during biodiesel synthesis. Fuel.

[146] Deus W.B., Ventura M., Silva J.R., Andrade L.H.C., Catunda T., Lima S.M. (2019). Monitoring of the ester production by near-near infrared thermal lens spectroscopy. Fuel.

[147] Gvozdev D.A., Maksimov E.G., Paschenko V.Z. (2020). Photobleaching of Phthalocyanine Molecules within a Complex with Colloidal Quantum Dots. Mosc. Univ. Biol. Sci. Bull..

