# Supplementary material for: Features of High-Precision Photothermal Analysis of Liquid Systems by Dual-Beam Thermal Lens Spectrometry
Source: Nanomaterials (Basel). 2024 Oct 1;14(19):1586. doi: 10.3390/nano14191586 (PMC11478039; doi:10.3390/nano14191586)
Supplement: Supplementary file 1 [file nanomaterials-14-01586-s001.zip › nanomaterials-3186127-supplementary.pdf]

Table S1. Parameters of dispersions of silicon oxide nanoparticles

Ludox	Average particle diameter d_{av}, nm	Concentration, % w/w	Density, kg/m^3	Specific surface, m^2/g
SM-30	7	30	1.220	350
HS-40	12	40	1.310	220
TM-50	22	50	1.400	140

Table S2. Parameters of dispersions of polystyrene nanoparticles

Nanoparticles	Av. particle size, nm	Functional groups	PDI	ζ-potential, mV	Concentration, % wt.
MD-10	80	$-SO_3$, –COOH	0.02	–72	0.025–0.15
MD-10-R	80				0.0005–0.003
MD-11	65	$-SO_3$, –COOH	0.03	–75	0.025–0.15
MD-11-R	65				0.0005–0.003

Table S3. Thermal-lens measurement parameters

Parameter	Value
Excitation laser	
Wavelength, λ_e (nm)	532.0
Focusing lens focal length, f_e (mm)	200
Confocal distance, z_{ce} (mm)	10.9
Laser power at cell, P (mW)	20–300
Spot size at the waist, ω_{e0} (μm)	42
Probe laser	
Wavelength λ_p (nm)	632.8
Focusing lens focal length f_p (mm)	300
Confocal distance, z_{cp} (mm)	2.7
Laser power at cell (mW)	4.5
Spot size at the waist, ω_{p0} (μm)	23
Spot size at cell, ω_p (μm)	90
Other constants	
Cell length (mm)	10
Sample-to-detector distance, z_2 (cm)	230
Mode mismatch factor m	4.59
Geometric parameters V	4.89
Modulator frequency (Hz)	0.25
Number of transient curves to average	300
Number of experiment repetitions	3–5
